# An Undergraduate Laboratory Module Integrating Organic Chemistry and Polymer Science

**DOI:** 10.1021/acs.jchemed.3c01194

**Published:** 2024-03-26

**Authors:** Arya Patel, Michael Arik, Amrita Sarkar

**Affiliations:** Department of Chemistry & Biochemistry, Montclair State University, Montclair, New Jersey 07043, United States

**Keywords:** Polymer Synthesis, Polymer Characterization, Upper-Division Undergraduate, Laboratory Instruction, Hands-on Learning, Communication/Writing

## Abstract

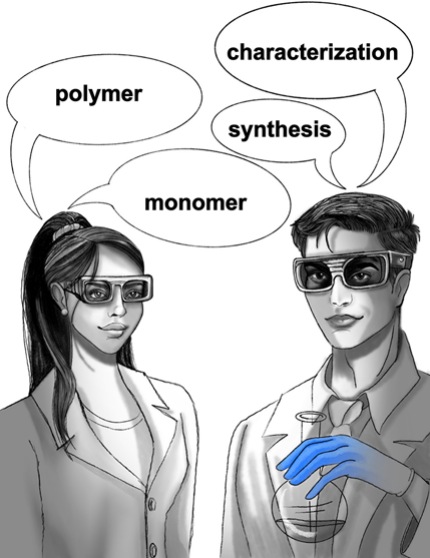

Polymer science is receiving wider acceptance in the organic chemistry community; thus, it is imperative to include it in the undergraduate organic chemistry curriculum. Despite the ever-increasing popularity of the topic of polymer chemistry in undergraduate curricula, a comprehensive laboratory experiment module describing a polypeptide synthesis by ring-opening polymerization of N-carboxyanhydride (NCA ROP) and a homopolymer synthesis by activators-regenerated by electron-transfer for atom transfer radical polymerization (ARGET ATRP) has yet to be proposed. Herein, we report a semester-long, ten week undergraduate laboratory module focusing on the synthesis and analytical characterization of polyalanine and polystyrene for an advanced organic chemistry class. Students received hands-on-experiences in synthesizing polymers followed by their characterization via proton nuclear magnetic resonance (^1^H NMR) spectroscopy, electrospray ionization-mass spectrometry (ESI-MS), gel permeation chromatography (GPC), thermogravimetry (TGA), differential scanning calorimetry (DSC), and transmission electron microscopy (TEM), which are not well-presented in the typical organic chemistry curricula. These engaging hands-on lessons in the newly designed laboratory module not only increase students’ interests in an interdisciplinary environment of organic chemistry and polymer science but also cultivate their research interests and communication skills and promote critical thinking.

## Introduction

Synthetic polypeptides and petroleum derived polymers are promising structured materials with remarkable functionality, which are used in a plethora of applications ranging from drug delivery and tissue engineering to nanotechnology. For example, polyalanine (PA), a biomolecule is not only relevant to the biomedicine^1^ but deeply impacts the area of molecule-based devices, such as magnetic memories and sensors.^2,3^ Thus, synthesis and characterization of PA is appropriate for teaching in an undergraduate chemistry laboratory.^4,5^ Likewise, polystyrene (PS), a commercially popular synthetic polymer is appropriate for chemical education,^6^ as its academic and industrial relevance increases day by day, due to its low cost and versatile applications in the packaging and building industries, as well having in domestic, medical, and automotive uses.^7−9^ Though synthetic polymers and polypeptides attract the interests of the scientific community, laboratory experiments focusing on their detailed synthesis and characterization are largely absent from the undergraduate chemistry curriculum.^10−13^ Considering the increased demand for polymer in both academia and industry, it will be valuable for undergraduates to learn the basics of polymer and acquire skills in synthesis and analytical characterization of it. Therefore, we launched a semester-long advanced organic chemistry laboratory module for upper-level undergraduates majoring in chemistry and biochemistry at Montclair State University. The experiments within this module explore the concepts of controlled ring opening and “living” free radical polymerizations and provide an opportunity for students to hone their laboratory skills. Moreover, students were guided through the working principles of advanced characterization techniques including gel permeation chromatography (GPC), transmission electron microscopy (TEM), thermogravimetric analyzer (TGA), and differential scanning calorimetry (DSC), which are not typically included in the traditional organic chemistry laboratory courses. These activities engage students in data collection and analysis. The overall laboratory activity outcome must contribute to students’ understanding of the fundamental chemistry to reach a target audience; thus, this laboratory course allows students to integrate their knowledge into a journal style lab report and communicate that to their peers via a classroom presentation.

## Pedagogical Goal

This lab was introduced in the Department of Chemistry and Biochemistry at Montclair State University in response to the call of the American Chemical Society (ACS) to include polymer science in the undergraduate curriculum. The main aim of introducing this comprehensive 10-week laboratory module was to encourage undergraduates to *think beyond small molecules*. Each experiment in this module leverages advanced synthetic routes, characterization methods, and data analysis to spur student engagement and satisfy the following learning goals:

(1) *Introduce the topic of N-carboxyanhydride ring opening polymerization (NCA ROP) to the students in lab*: NCA ROP is widely used in synthesizing polypeptides in large scale with controlled molecular weights and high optical purity.^14,15^ Undergraduates are typically introduced to the ring structure of NCA, its opening by nucleophilic attack and subsequent chain growth by polymerization in the introductory organic chemistry lectures. This laboratory course offers a platform to translate that acquired theoretical knowledge into practice.
(2) *Introduce the topic of activators regenerated by electron transfer for atom transfer radical polymerization (ARGET ATRP)*: ARGET ATRP is an important class of controlled “living” radical polymerization, commonly used by industry and academic community. Though free radical reaction and its role in commercial polymer synthesis is covered in the organic chemistry lectures, students in undergraduate laboratory often do not receive the opportunity to gain experiences in preparing polymer via radical polymerization. This present laboratory module is developed to provide students an opportunity to receive hands-on experiences in this robust technique.
(3) *Introduce a range of analytical instruments*: To apprehend students’ attention, this course is designed to introduce several popular analytical instruments for polymer characterization including GPC, TGA, DSC, and TEM, which are not covered in the traditional undergraduate organic chemistry curriculum.
(4) *Data analysis*: Interpreting data is one of the major practices in science education. Hence, in parallel with the lab activities, students are introduced to advanced data analysis techniques. For example, students are taught the ^1^H NMR end-group analysis for determining the number-average molecular weight (*M*_n_) and % chain end functionality of a homopolymer. Likewise, they are also taught to analyze electron microscopy images (e.g., TEM and ImageJ software) to estimate the size of self-assembled polypeptide aggregates.
(5) *A formal lab report and an oral presentation*: The final goal of this lab is to acquaint students with writing a formal lab report following standard ACS formatting guidelines and effectively communicate that through an oral presentation (PowerPoint presentation). All the designed experimental learning goals are summarized in Table 1.

**Table 1:** Learning Goals

Item	Learning Goals
1	Students will develop their skills in polymer synthesis via NCA ROP, and ARGET ATRP.
2	Students will conduct ESI-MS analysis, ^1^HNMR end-group analysis, and GPC profile interpretation to determine polymer molecular weights and dispersity.
3	Students will analyze thermal stability of polymers by TGA and DSC.
4	Students will learn the concept of self-assembly and determine size of self-assembled aggregate nanostructures by TEM image and Image J software.
5	Students will be familiarized to the ChemDraw software
6	Students will sharpen their communication skills by conveying the conclusions of each laboratory task to their peers through an organized class discussion and writing a scientific lab report in an ACS journal style.

## Laboratory Experiment Overview

This 10-week laboratory module was offered in Spring 2023 and designed as a critical and mandatory component for an advanced organic chemistry elective course (CHEM 490). Participating students have completed organic chemistry I and II sequence lectures and laboratories (CHEM 230/231/232/233). Ten senior undergraduates enrolled in this course, divided into 3 groups, met weekly with the instructor in a 3 h lab period to conduct experiments, synthesized and characterized polymers, analyzed data using learned techniques and software, and participated in an instructor-led group discussion. Each week lab activity is summarized in Table 2.

**Table 2:** Week-Specific Laboratory Activity Details

Week	Laboratory Activity
1	Polyalanine (PA) synthesis via NCA ROP
2	Purification of PA
3	ESI-MS sample preparation and introduction to Chem Draw software. Students learned the working principle of ESI-MS and the use of ChemDraw software in drawing molecules and analyzing their masses
4	ESI-MS data analysis for PA. Students were able to analyze the ESI-MS data with the help of ChemDraw software drawn molecules and convey the conclusion to their peers through an organized class discussion
5	TEM, TGA, and DSC sample preparation for PA and introduction to Image J software
6	(i) TEM, TGA, and DSC data analysis for PA. Students were able to analyze the TEM data using Image J. They also analyzed the TGA/DSC thermogram to determine degradation temperature for PA. (ii) Polystyrene (PS) synthesis start
7	(i) PS purification (ii) ^1^H NMR, GPC, TGA, and DSC sample preparation for PS.
8	^1^H NMR data analysis for PS. Students learned how to determine the number-average molecular weight (*M*_n_) and % chain-end functionality for PS by ^1^H NMR end-group analysis.
9	GPC, TGA, and DSC data analysis for PS. Students gained familiarity with GPC analysis. They learned the concepts of glass transition temperature and determining it from the collected DSC thermogram.
10	Students submitted final lab report describing all lab activities, and corresponding data and final PowerPoint presentation

Because of the lack of some analytical instruments on the university campus (for, e.g., GPC, TGA, and DSC), these characterizations were conducted by the instructor in external research facilities, and the individual raw data (in excel format or/and chromatogram/thermogram) were distributed to each student team for analysis. During each week, students were provided handouts describing week-specific activity (section 2, 10 handouts, Supporting Information). Students attended a 45 min lecture session separately, in which the instructor presented an overview of the reaction/experiment, working principle of the analyzing instrument/software, and data analysis strategy. Finally, the students presented their lab work through a literature discussion, scientific lab report, and PowerPoint presentation. Lab report template is described in section 3, Supporting Information. Two take-home exams were arranged (section 5, Supporting Information) to examine students’ understanding of the topics covered in this course. Overall, this module was divided into 3 laboratory activities, described below.

### Activity 1. Synthesis and Molecular Characterization of Polyalanine (PA)

**Scheme 1:**
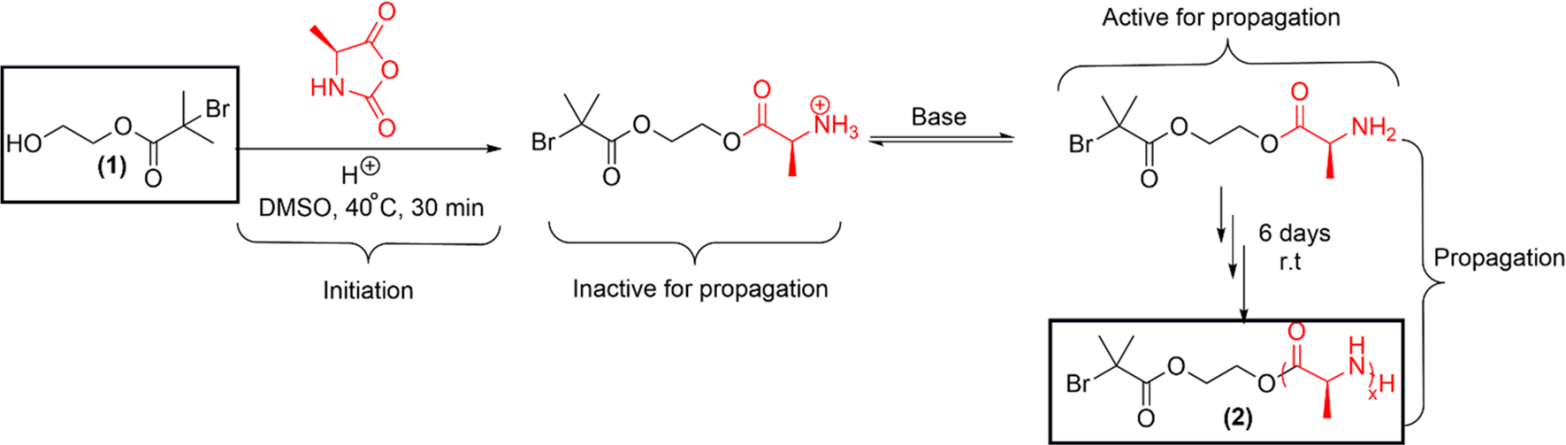
Proposed Synthesis Scheme for Polyalanine (PA) **(2)** via NCA-ROP

Typically, uniform and sequence-controlled polypeptides are synthesized by solid-phase peptide (SPP) method and have been implemented in a number of undergraduate educational teaching laboratories.^16−18^ Despite success, repetitive protection/deprotection cycles and the requirement of excess solvents for washing and a large molar excess of reagents limit the use of cost-prohibitive SPP in teaching method. On the contrary, a low-cost N-carboxyanhydride ring opening polymerization (NCA ROP) receives wide appreciation in synthesizing polypeptides in large scale.^14,15^ Though several undergraduate laboratory exercises have been designed to synthesize architecturally advanced polymers via ROP,^11,19,20^ an undergraduate laboratory experiment focusing on the synthesis of polypeptide via NCA ROP is still limited. Considering this, a 5-week laboratory module was designed where students synthesized PA via NCA ROP followed by conducting its molecular characterization, thermal analysis, and solution self-assembly. In the prelab lecture, the topic of ROP was reviewed with the required information. Such as, students were informed that the traditionally primary amines are used as initiator to synthesize polypeptides from NCA due to its fast rate of initiation and efficient propagation steps.^14,21−23^ Conversely, hydroxyl group (OH) initiated NCA-ROP shows significantly slower rate, and thus yields poorly defined polypeptides, though it works well on epoxide, lactone, and cyclic carbonate monomers.^11,19,20^ However, Pahovnik and co-worker reported an acid-catalyzed OH-initiated controlled NCA ROP, that overcomes the slow initiation step and leads into well-defined polypeptides and peptide-based block copolymers.^24^ Following this approach,^24^ students synthesized PA using an initiator 2-hydroxyethyl-2-bromo isobutyrate **(1)** (contains a primary alcohol functional group) and a catalytic amount of methanesulfonic acid (MSA), shown in Scheme 1. Students learned that the MSA assisted initiator not only catalyzes the alanine NCA ring through its OH group but also protonates the amine, which further reduces the chance of uncontrolled chain propagation. Propagation starts only after the deprotonation of the ammonium group in the presence of a base *N*,*N*-diisopropylethylamine (DIEA).

**Figure 1:**
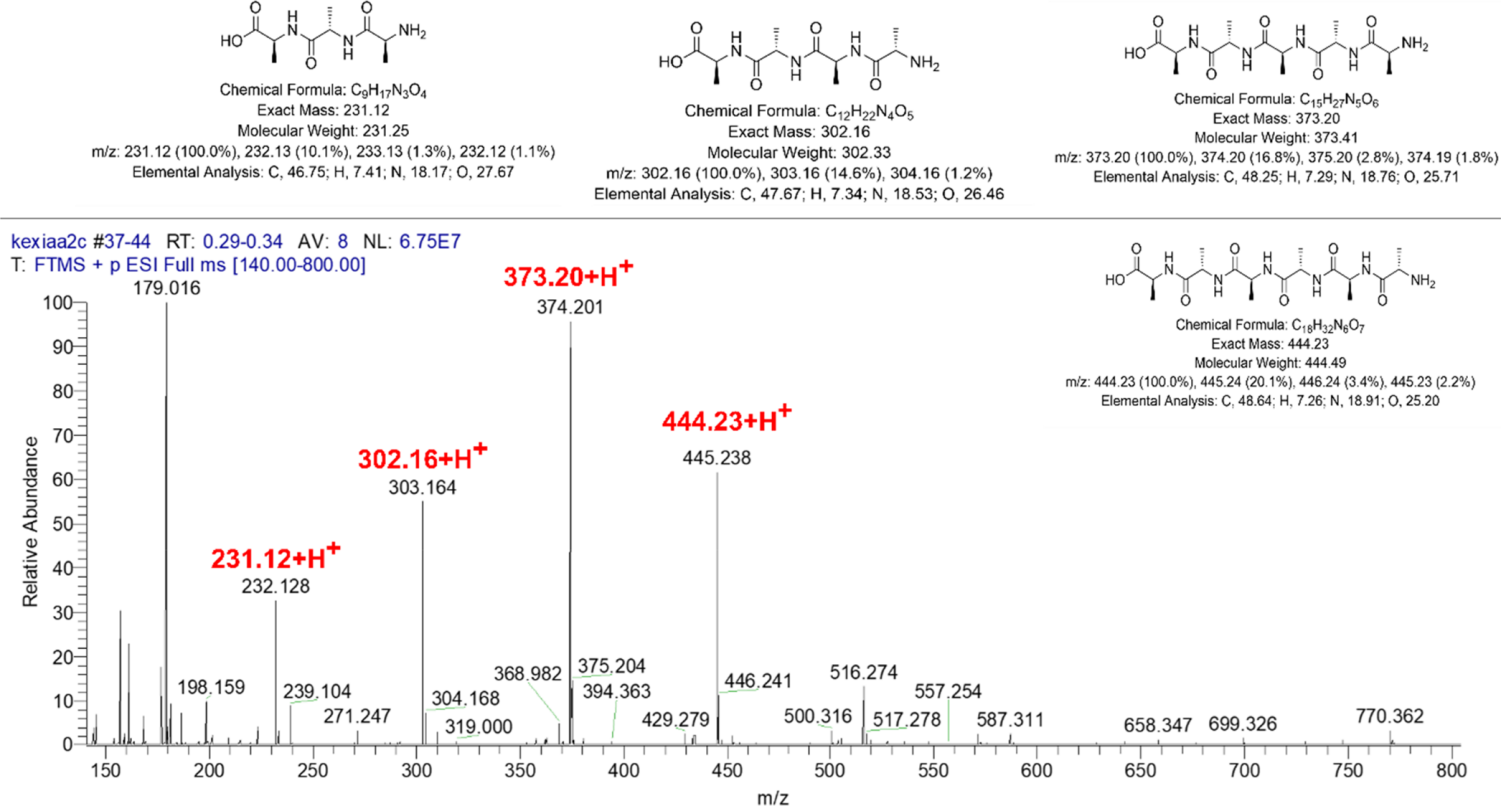
ESI-MS with denoted measured monoisotopic signals of a PA, synthesized by a representative student team using alanine N-carboxyanhydride and initiator **(1)**, as proposed in Scheme 1.

Students performed the synthesis and purification of PA in the first 2 weeks laboratories; detailed experimental condition and purification procedure are described in section 2, Handout 1–2, Supporting Information. The isolated and purified products (Figure S6) were characterized by electrospray ionization mass spectrometry (ESI-MS), described in the result section (Figure 1). Fundamentals and working principle of mass spectrometry (MS) are commonly taught in the introductory organic chemistry lecture courses. Students were introduced to the use of MS in PA repeat unit identification (section 2, Handout 3–4, Supporting Information) during the third and fourth week of laboratory activities. Students were encouraged to draw possible PA structures with varying repeat units and to deduce their molecular masses. In consequence, they were introduced to ChemDraw, a licensed software commonly used for drawing chemical structures. A few representative student-drawn PA structures are shown in Figure 1.

### Activity 2. Synthesis and Molecular Characterization of Polystyrene (PS)

**Scheme 2:**
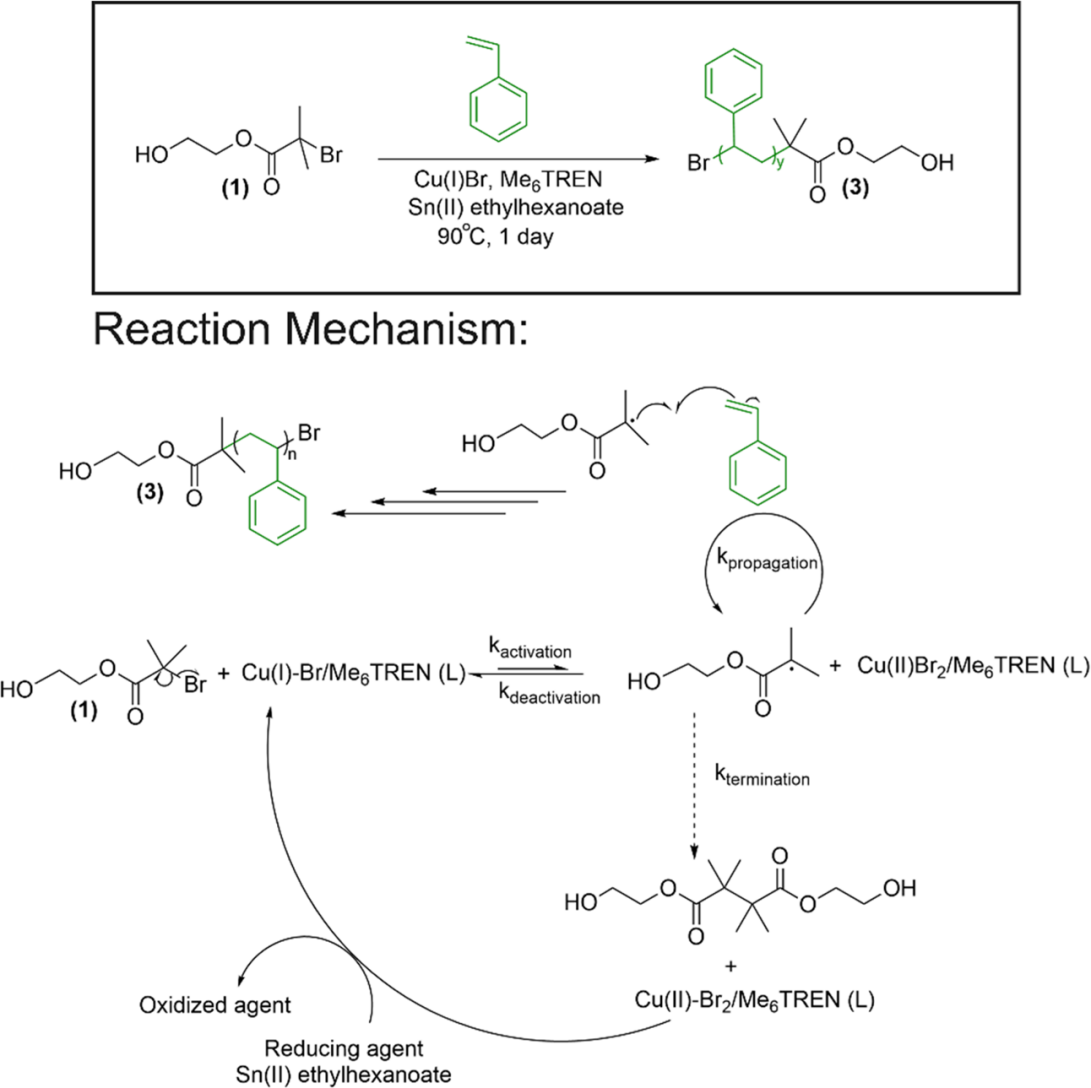
Proposed Synthesis Scheme for PS **(3)** via ARGET ATRP. Student drawn reaction mechanism is shown.

In Activity 2, students synthesized a well-defined homopolymer PS via ARGET ATRP using the same dual functionalized initiator **(1)** containing an alkyl halide at the other end, proposed in Scheme 2. To the best of our knowledge, no undergraduate laboratory experiments describing PS synthesis by ARGET ATRP have been reported. Instead, wide reporting of atom transfer radical polymerization (ATRP) in undergraduate teaching laboratory^25−27^ we choose the method as ARGET ATRP, primarily for three reasons (vide infra).

**Figure 2:**
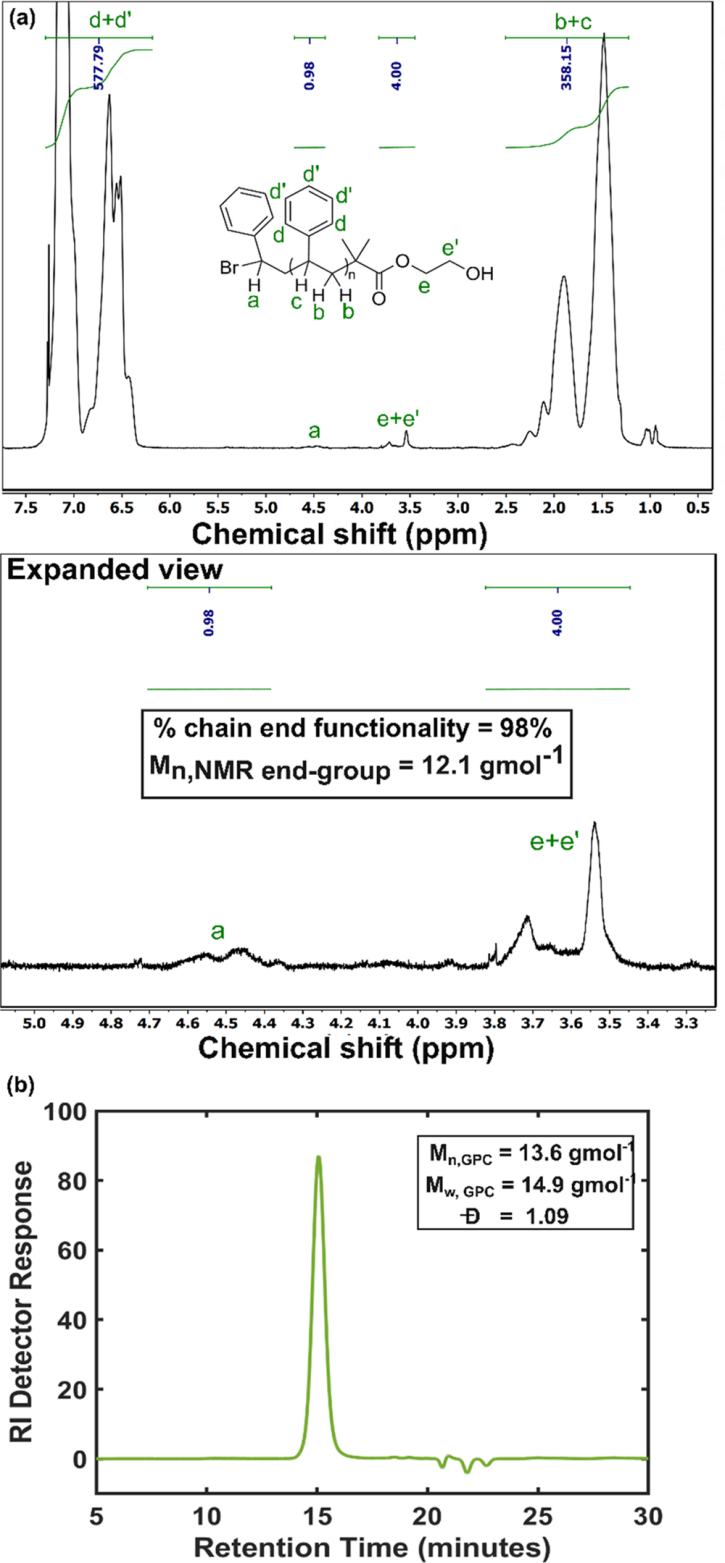
^1^HNMR spectrum of PS (recorded in CDCl_3_), synthesized by a representative student team via ARGET ATRP (proposed in Scheme 2) is shown in (a). A strong peak at 7.26 ppm was found due to residual CHCl_3_ in the NMR solvent. GPC of the same PS is shown in (b).

(1) ARGET ATRP is advantageous as it involves a significantly lower amount of copper (Cu) based active catalyst compared to classical ATRP.^28^ A constant regeneration of the Cu (I) activator species facilitates in this process in the presence of a reducing agent tin(II) ethylhexanoate, which reacts with the deactivator Cu(II) (usually generates during the termination reaction) and converts back to the activator Cu(I). Thus, a high monomer conversion using a minute catalyst concentration could be achieved via this greener approach. Moreover, the presence of reduced level of copper in the resultant PS simplifies the purification process. Students recovered their product simply by dissolving the crude mixture in tetrahydrofuran and precipitating that in chilled methanol (Figure S4), whereas polymers synthesized by ATRP typically requires alumina column purification before precipitation step to remove copper contaminant from the product.^29,30^
(2) ARGET ATRP shows improved tolerance of oxygen compared to ATRP and simplifies the polymerization procedure. Traditionally, ATRP is carried out under an inert atmosphere with three-to-four successive freeze–pump–thaw (FPT) cycles to ensure the complete elimination of residual air from the reaction flask. However, FPT requires Schlenk line setup with specialized gas manifold, vacuum system, and cryogenic liquids that is not always present in teaching laboratories at smaller institutions. Instead of FPT cycles, students degassed their reaction flasks with nitrogen gas for 15 min in the current experiment and obtained PS with controlled molecular weight and narrow dispersity (see in Figure 2).
(3) The styrene chain end functionality is often lost during ATRP due to the side reaction between copper catalyst and growing chain and thus limit producing PS with high chain end-functionality.^28,31^ Students learned that high chain end functionality in a homopolymer is crucial to have to synthesize further a well-defined polymer (e.g., block copolymer). ARGET ATRP lowers the possibility of β-hydrogen elimination or other side reactions remarkably and produces PS with controlled chain length, dispersity, and improved chain end functionality.

Consolidating these, we designed another 5-week laboratory activity to synthesize PS with target degree of polymerization (DP) of 150, adopting the procedure from preciously published PS synthesis via ARGET ATRP.^32^ The weeks of 6 and 7 were dedicated to this lab activity; detailed synthesis/purification procedures along the safety considerations are described in section 2, Handout 6–7, Supporting Information. The concept of “living” and controlled radical polymerization, DP, and molecular weights such as number-average (*M*_n_), weight-average (*M*_w_), and dispersity (*Đ*) were discussed in the preceding lectures. The synthesized PS (Figure S7) was characterized by ^1^H NMR and GPC, described in the result section (Figure 2). Small molecule characterization by liquid NMR spectroscopy is an integral part of the introductory organic chemistry curriculum. However, characterizing polymer via NMR end-group analysis is not usually studied by chemistry undergraduates, though it is an important skill to be acquired.^25^ Therefore, a discussion was dedicated with special focus on determining the molecular weight of PS by NMR end-group analysis. Each student team analyzed their obtained NMR spectra and learned the importance of manual phase and baseline corrections and differences in T1 relaxation rates, while analyzing accuracy of a signal integration. A student work in determining *M*_n_ by NMR end-group analysis is described in result section and in section 4, Supporting Information. Similarly, GPC is not usually included in organic chemistry laboratory curriculum, but widely employed in polymer and biochemistry laboratory experiments, due to its ability to determine the length and dispersity of the macromolecules.^33^ Introduction to GPC through this laboratory course allows students to learn another chromatography technique. They gain an understanding of a principle that GPC works based on the size only; that is, a heavier chain elutes first (lower retention time) as it interacts with the pores in the stationary column the least. On the other hand, smaller chain spends the maximum time inside the pores, thus retains and elutes at the last at higher retention time. Students analyze the GPC profile for their synthesized PS and conceptualize their findings within the fields of polymer, organic chemistry, and chromatography separation.

### Activity 3. Thermal Properties and Self-Assembly

**Figure 3:**
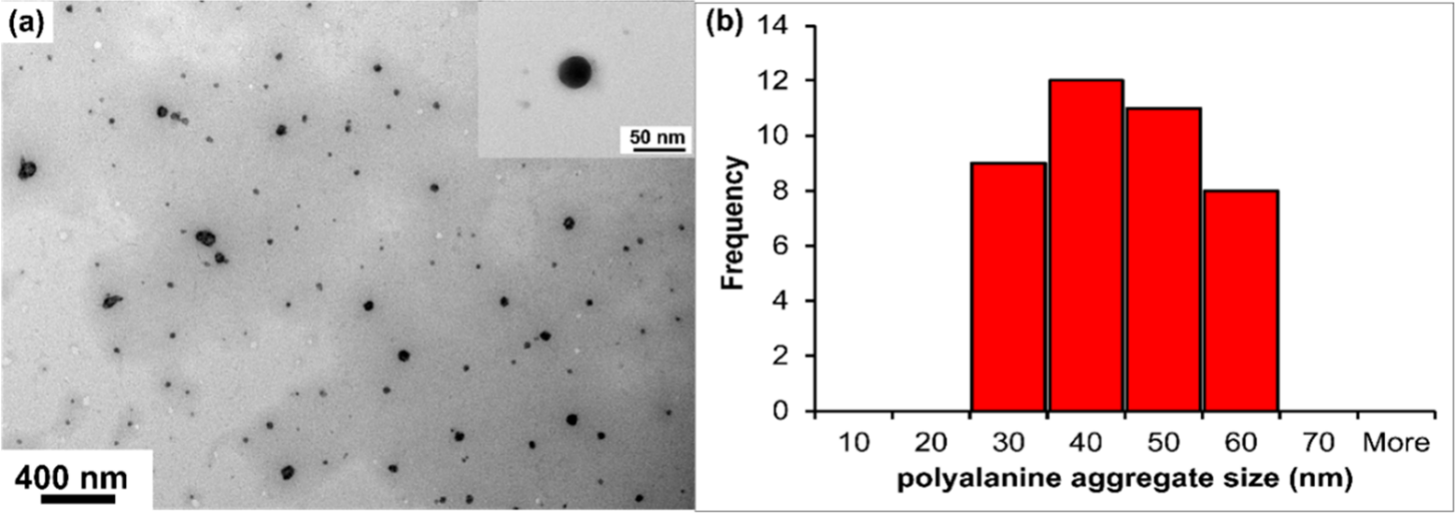
TEM image (a) of a representative PA self-assembled nanostructure in aqueous medium and its size distribution (b). A uniform spherical aggregate structure is inscribed above (a). The TEM image was obtained on a Hitachi H-7500 tungsten/LaB6 TEM operated at 120 keV accelerating voltage.

**Figure 4:**
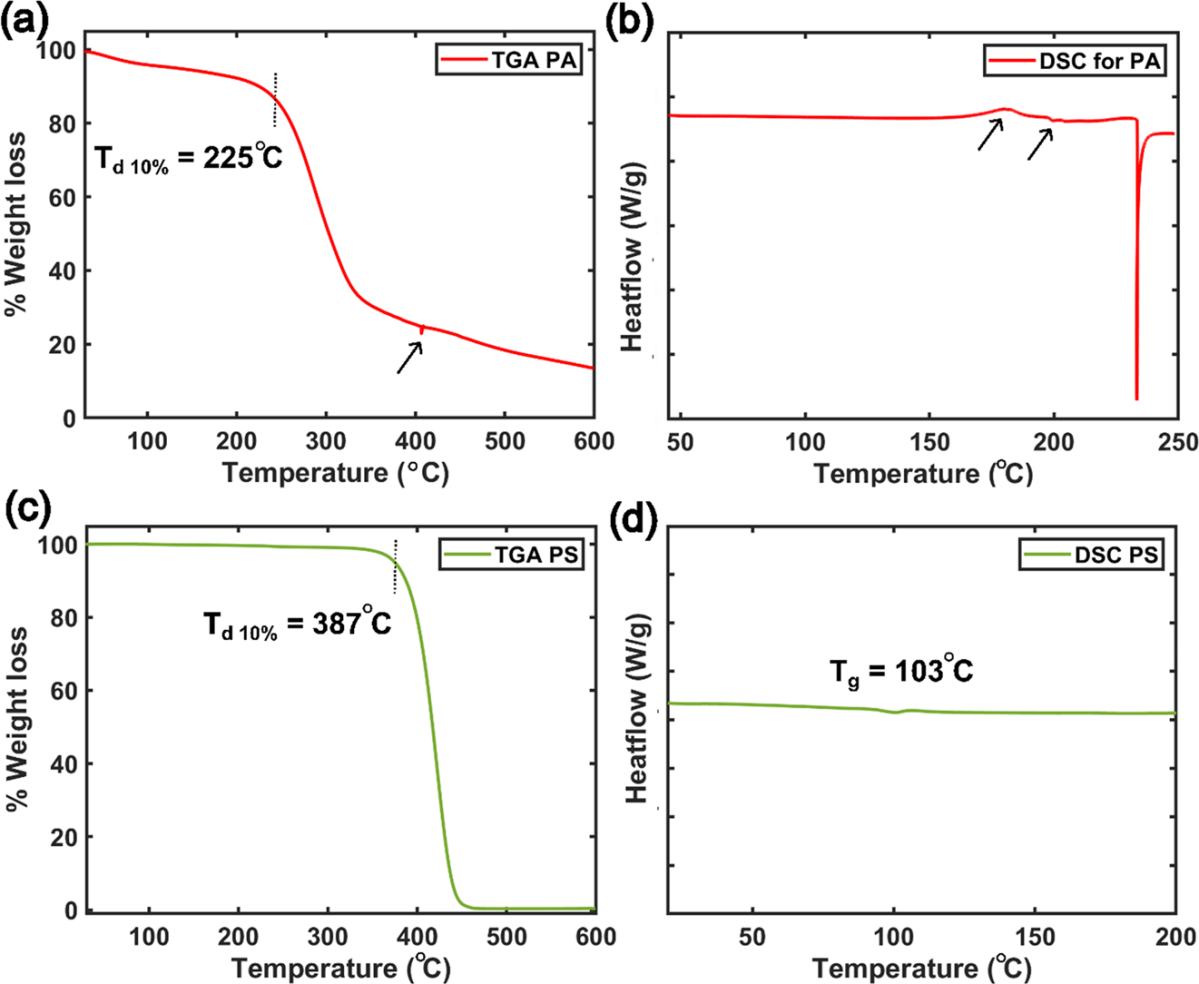
TGA and DSC thermograms of PA (a, b) and PS (c, d). Artifacts are shown by arrows. The decomposition temperature (*T*_d_) was measured by identifying the point in the TGA profile, where 10% of the total weight was lost.

Although molecular characterization techniques, including ^1^HNMR, GPC, and ESI-MS, remain relevant, students substantially benefit from learning new techniques such as electron microscopy and thermal analysis. Exposure to these techniques becomes advantageous for students, especially those who continue their career in industry and broader range of academic programs in graduate schools including chemistry, chemical engineering, and material science.^34^ Considering this, weeks 5, 6, and 9 were assigned to the lab activities associated with TEM, TGA and DSC for the synthesized PA and PS. Sample preparation details and results are described in section 2, Supporting Information and in Figures 3 and 4, respectively. Motivation for this development was 3-fold: (i) familiarize students to the working principles of the proposed instruments, (ii) train them in produced image or data analysis, and (ii) teach in the concepts of self-assembly and thermal stability of macromolecules. Peptide self-assembles into well-defined hierarchical structures that have received considerable attention in biological systems and in biomedical and biomaterial fields. For example, aggregation behavior of PA is often studied for understanding human disease mechanism.^1^ Moreover, advanced nanostructures obtained from the polypeptide and peptide conjugates are successfully used in drug delivery.^35^ It is therefore necessary to introduce the concept of peptide self-assembly and include a microscopic observation of the self-assembled structure in the undergraduate chemistry laboratory curriculum. Similarly, students were introduced to the concept of the glass transition temperature (*T*_g_) and use of DSC to measure it. The *T*_g_ for a polymer is defined as the temperature below which the polymer transitions to a hard, glassy, or brittle state from a soft and rubbery or ductile phase. Students were also introduced to TGA to determine the decomposition temperature (*T*_d_) and optimum thermal stability of a new polymer material.

## Hazards

The laboratory must be equipped with a fire extinguisher. All syntheses and purifications must be performed in well-ventilated fume hoods. All of the reactions must be carried out under the supervision of the instructor. Every reagent including styrene, tris-2-dimethylaminoethyl amine (Me_6_TREN), tin(II) ethylhexanoate, Cu(I)Br, methanesulfonic acid, 2-hydroxyethylbromoisobutyrate, acetonitrile, dimethyl sulfoxide (DMSO), tetrahydrofuran, and methanol should be handled inside hood with care. Before students handle these chemicals, they should be provided with comprehensive information about their toxicity, flammability, potential hazards, and proper handling procedure. Students wear goggles and gloves throughout the lab activities. Students should be cautioned regarding the use of syringes and needles. Liquid and solid waste should be disposed of in properly labeled and sealed containers following health and safety guidelines. Material Safety Data Sheets (MSDSs) are freely available in the lab. Please refer to the *handouts* in the Supporting Information for more specific safety-related information.

## Results and Discussion

Lab experiment began with Activity 1, where students synthesized PA via NCA ROP method following Scheme 1 (procedures provided in Handouts 1 and 2) and characterized that by ESI-MS, shown in Figure 1.

Careful analysis of all three-team produced ESI-MS data and possible product structural analysis reveal that the ROP occurs successfully, and the resultant product is a combination of polydisperse alanine with repeat units of 3–6. However, all three teams produced PA chains that carried the primary amine group on one side and not attached with the desired protected group on the other end (as proposed in Scheme 1), rather terminated with a free hydroxyl group (Figure 1). Students performed a literature search^36,37^ to explain their data and attributed this phenomenon to the predominant initiation of alanine NCA by water instead of the initiator **(1)**. Water might come from insufficiently dried glassware, moist surroundings, and wet reagents/solvents (stored in a laboratory cabinet without any protection gas) and is unable to be eliminated completely by a constant flow of nitrogen gas. This result allowed students to think critically that controlling the trace level of water during NCA ROP is crucial for obtaining well-defined and accurate end-functionalized polypeptides. The NCA ROP is usually performed under an inert atmosphere (e.g., glovebox) in a research laboratory to avoid water contamination. Therefore, a modification will be applied to this task in the future where this reaction will be performed in a glovebox and all the reagents will be stored in glovebox, as well. Students synthesized PS in Activity 2 via ARGET ATRP (Scheme 2, procedure in Handouts 6 and 7) and characterized that by ^1^H NMR and GPC, shown in Figure 2. Students identified broad peaks in NMR that are responsible for aromatic (assigned **d** and **d′**), and aliphatic regions (assigned **b** and **c**) of the repeating monomer unit of PS. They also identified the signal of initiator methylene proton (−CH_2_, assigned **e** and **e′**) at 3.50–3.80 ppm. Finally, the signal at 4.35–4.65 ppm was assigned to proton (a) on the carbon adjacent to bromine of the growing PS chain. Students’ attention was drawn to the fact that no trace of signal was found at the 6.0–6.3 ppm which suggests that the termination reactions including β-hydrogen elimination and bimolecular disproportionation both were suppressed significantly in the described ARGET ATRP reaction.^28^ Each team performed ^1^H NMR end-group analysis of the acquired spectrum. The *M*_n_ estimated by a representative student team is reported to be 12.1 g mol^–1^ with 77% monomer conversion and excellent chain end functionality of 98% (Figure 2a), please see the detail student work in section 4, Supporting Information.

All three teams estimated the DP for their synthesized PS samples in the range 110–130, while the target DP was set at 150. This laboratory task was a demonstration to the students that retention of active Br chain end-functionality could be achieved via ARGET ATRP in a moderately high molecular weight homopolymer with a high monomer conversion and minimum chance of side reactions, which makes it an appropriate living candidate for further use in a block copolymer synthesis. The *M*_n_ obtained by ^1^H NMR was found to be close to the GPC estimated value of 13.6 g mol^–1^ and low *Đ* (*M*_w_/*M*_n_) of 1.09 (Figure 2b). The low dispersity indicates a narrow distribution of PS chain length that confirms the efficacy of the students’ performed ARGET ATRP. Two student teams were successful in producing monomodal and low dispersed (*Đ* < 1.10) PS; however, one team exposed their synthesis to the air during handling Schlenk flask and obtained PS with broader dispersity (*Đ* > 1.3). Additional characterization techniques such as infrared (IR) (Figures S10 and S11) could be added in this module in the future along with ^1^NMR and GPC. A future modification can be applied on PS synthesis using low-cost reagents such as the *N*,*N*,*N*,*N*,*N*-pentamethyldiethylenetriamine (PMDETA) ligand and ascorbic acid reducing agent (section 7, Figure S13, Supporting Information), which may allow institutions to involve more students in such experiments at lower cost.

Finally, in Activity 3, students analyzed the aqueous self-assembly of PA, evaluated by TEM. A representative image from a student group is shown in Figure 3 that exhibits a distribution of uniform spherical self-assembled PA aggregates. Students determined the average hydrodynamic dimeter of these spherical structures by ImageJ analysis^38^ and reported the diameter in the range of 30–60 nm (average aggregate size = 38.6 nm and standard deviation = 9.5 nm (total sample size = 40). This result was a demonstration to the students that sequence and length-controlled synthetic peptide repeat unit forms well-defined nanoscale morphology, like the repeat sequences regularly found in natural proteins.^1,21,39^ Lastly, students performed thermal analysis of these two synthesized polymers, as shown in Figure 4. TGA measurement for PA sample (Figure 4a) showed 10 wt % decomposition (*T*_d,10%_) at 225 °C, which reflects the degradation-induced mass loss. Whereas the DSC thermogram for PA was featureless (Figure 4b) except for a huge endotherm around 233 °C, probably due to the overlapping of melting and thermal degradation of PA. A few sharp endothermic peaks (shown by arrow) were noticed in all three-student teams produced DSC thermogram at nonreproducible temperatures, which are possibly artifacts. Students performed literature search to explain this observation and found that artifacts may originate by the fluctuation of powder samples or the sample holder in the DSC sensor due to the effect of thermal expansion.^40^ Students evaluated the thermal properties of the PS, as well. They measured the *T*_g_ by taking intersection of the extrapolation of the baseline with the extrapolation of the inflection in DSC profile of PS (Figure 4d). They identified *T*_g_ at 103 °C and concluded that PS begins to flow freely at this temperature due to its chain mobility. On the other hand, the TGA profile for PS shows 10% weight loss temperature at 387 °C (Figure 4c). Students are attracted to the thermal analysis lab as they get an idea of the applications for which their synthesized polymer candidates are appropriate. Later, students were asked to compile all the collected experimental data, write a lab report, and prepare a PowerPoint presentation with detailed discussion. This comprehensive guided-inquiry laboratory module motivates students to think more critically about their learned chemistry. We expect this current work will contribute to the recent developments^20,30,41−44^ in implementing polymer synthesis and characterizations into undergraduate teaching laboratory.

### Student Assessment and Evaluation

The effectiveness of this lab module was assessed based on the data obtained from the grading rubric (section 6, Supporting Information) and performance statistics (Table 3, Table S1). Students’ success was measured by evaluating their participation in the laboratory activities, organized class discussions, grading formal lab reports, grading two take home exams, and the final oral presentations. For example, the average score for the category of “Students’ Lab Performance” was 90%; points were deducted for insufficient contribution to the lab activity while working in a team or for inability to explain the reaction principle/mechanism, instrument working principles, and not following the safety rules. Likewise, the average score for the category of “Formal Lab Report” was 85%; most laboratory reports were well-written, students incurred point deduction primarily for incorrect calculation of *M*_n_ from ^1^H NMR end-group analysis, inadequate explanation for *T*_g_ and *T*_d_ from thermal analysis data and incorrect reference style, etc. The category of “Oral Presentation” evaluates students’ communication skills, where each team delivered an oral talk for 10 min describing their finding from these 10 weeks lab activity and necessary literature discussion. This category was evaluated by their presentation style and content of the slides, and the class average was found as 86%. The “Take Home Exam” average was 80%, which confirms that the students’ knowledge in polymer synthesis and characterization is greatly broadened. At the conclusion of the class, we found that most students are comfortable in describing the principles for proposed NCA ROP and ARGET ATRP reactions, challenges, and strategies to overcome those. Students were also proficient in describing the working principles of polymer characterization techniques, such as GPC, TGA, DSC, and TEM. They are also able to explain the mass spec data for different polypeptides, analyze polymer molecular weights by the ^1^H NMR end-group and GPC, and use ChemDraw and ImageJ software in drawing molecules and image analysis, respectively. A few participating students expressed strong interest in polymer related research and education.

**Table 3:** Assessment Score of Participating 10 Students

Materials to be graded	Assessment metrics	Students’ performance class average (%)
Students’ lab performance	(i) Read assigned literature thoroughly	90
	(ii) Be able to carry out each experiment following the protocol described in lab handout	
	(iii) Following safety rules properly	
	(iv) Following all the required steps with high accuracy during software use, and data analysis	
	(v) Participate in data discussion session with all other team members, peers in the class and the instructor	
Formal lab report	(i) Correct presentation of software drawn molecules (e.g., ChemDraw)	85
	(ii) Correct representation of self-assembled aggregate size (average and standard deviation) measured by a software (Image J)	
	(iii) Correct incorporation of all the experimental procedures with noting any changes performed during the experiment	
	(iv) Report all accurate data/spectra (HNMR/GPC/TGA/DSC) with proper label/instrument operation or measurement condition	
	(v) Provide reasonable explanation to the data/results	
	(vi) Following lab report guideline properly	
	(vii) Provide properly formatted reference (ACS style)	
Take home exams	Answer questions with satisfactory explanation	80
Oral presentation	Communicate the lab outcome (individual team data with literature discussion) in a scientific manner (PowerPoint)	86

In summary, we can say that we achieved the main goal of this laboratory module which was set originally. At the end of the course, students were given an opportunity to evaluate the laboratory module and provide feedback and comments in a course evaluation survey. All 10 participants found this semester-long laboratory course to be an enjoyable and valuable learning experience, which inspired them to think beyond small molecules. The majority of the class (90%) agreed strongly that the course offered relevant scientific information and technical skills. Students commented: “*I enjoyed learning about techniques that are not commonly used in a typical organic chemistry class”; I liked that the class has a lot of relevance to the industry and modern technology*”; and “*I think this course should be offered again for students in the future.”*

## Conclusion

Implementing an undergraduate organic chemistry teaching lab focused on the key concepts of polymer education is a significant matter that is addressed via this current effort. The present comprehensive laboratory exercise provides undergraduates an opportunity to use a multifaceted approach spanning from synthesis to characterization of polymers, analyzing their thermal stability and evaluating self-assembled nanostructure employing a variety of different analytical tools. Additionally, students gain the experience of documenting their laboratory work in a scientific journal style and communicating that orally. In summary, students at Montclair State University receive their first formal training in macromolecule or polymer synthesis and characterization. The challenging aspect of this newly implemented laboratory module was not receiving hands-on experiences in advanced characterization techniques, such as GPC, TGA, and DSC, as they are not located on the university campus. There are many opportunities to extend this new teaching laboratory in future; for example, the same experiment could be adapted to the introductory organic chemistry laboratory in second year by restricting it to the synthesis and basic characterization (^1^H NMR, MS). Or the combination of ROP and ARGET ATRP could focus on synthesizing architecturally advanced linear and brush-type block copolymer, which could be extended for advanced polymer laboratory class or an inquiry-based project.

## Supporting Information Available

Student handout, lab report guideline, additional students’ data, take home exams, and grading rubrics (PDF)
