# Supplementary material for: An Undergraduate Laboratory Module Integrating Organic Chemistry and Polymer Science
Source: J Chem Educ. 2024 Mar 26;101(4):1686–95. doi: 10.1021/acs.jchemed.3c01194 (PMC11008100; doi:10.1021/acs.jchemed.3c01194)
Supplement: Supplementary file 1 — ed3c01194_si_001.pdf [file ed3c01194_si_001.pdf]

Supporting Information

An Undergraduate Laboratory Module Integrating Organic Chemistry and Polymer Science

Arya Patel, Michael Arik, Amrita Sarkar *
Department of Chemistry & Biochemistry, Montclair State University, Montclair, NJ 07043, USA.
**Corresponding author:* sarkara@montclair.edu

Section 1. Materials………………………………………………………………...S1
Section 2. Lab Description, Instructor Note and Experimental Procedure…….S1-S12
Section 3. Lab Report Guideline………..…………………………………...S12-S13
Section 4. Students Data and Performances……………………..………….S13-S16
Section 5. Take Home Exams……………………………………………….S17-S26
Section 6. Grading Rubrics…………………………………………………..S26
Section 7. Additional Note…………………………………………….…S27-S32

Section 1. Materials

Reagents were purchased and used as received without further purification. Alanine N-carboxyanhydride (Ala NCA) or (S)-4-methyl-2,5-oxazolidionedione (CAS No. 2224-52-4) was purchased from LinkChem Co., Ltd. (Shanghai, China) and used as received. Dimethyl sulfoxide (DMSO, LC-MS grade, 99.9%, Thermo Fisher), methanesulfonic acid (99%, Acros Organics), 2-hydroxyethylbromoisobutyrate (96%, Aldrich), Diisopropylethylamine (>99%, TCI america), diethyl ether (BHT stabilized, Fisher), styrene (Stabilized by styrol, Acros), copper(I) bromide (99%, Thermo Fisher), Tin (II) ethylhexanoate (92%, Sigma Aldrich), tris-2-dimethylaminoethyl amine (Me_6TREN) (98%, Thermo Fisher), tetrahydrofuran (>99.5%, anhydrous, Acros), methyl alcohol (Fisher), acetonitrile (Fisher).

Section 2: Students Handout

Handout 1

Laboratory 1: Polyalanine Synthesis via N-carboxyanhydride Ring Opening Polymerization (NCA ROP)

Note: Please read the below article. Write down the initiation and propagation ratio in your notebook, proposed in the article. Experimental procedure described in this paper should be written in your notebook. Annotate any deviation or changes that you will do in this experimental procedure in this week's lab. Justify your changes to the formal lab report, which is due at the end of this course.

Reference Literature:

Gradisar, S.; Zagar, E.; Pahovnik, D. Ring Opening Polymerization of N-Carboxyanhydrides Initiated by a Hydroxyl Group. *ACS Macro Lett*. **2017**, *6*, 637-640.

Synthesis procedure:

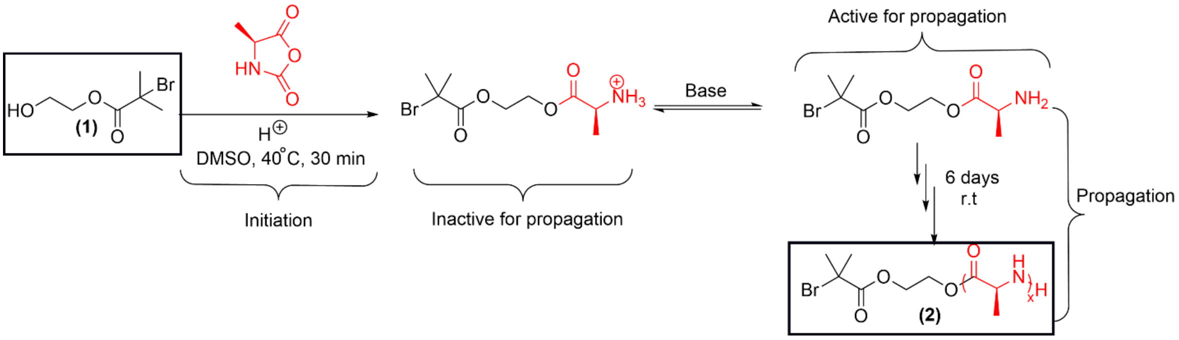

Each team should take one flame dried Schlenk flask equipped with a stir bar and go to the assigned hood space. You should add 2 g NCA-ALA (17.4 mmol) and 20 ml DMSO in that flask. The flask will be purged with nitrogen gas (**Follow Instructor's direction**: HOW TO ADJUST the PRESSURE ON A NITROGEN GAS CYLINDER REGULATOR) and stir for 10 minutes to get a well dispersed solution. Next, you should add 124 µL (0.872 mmol) of the initiator 2-hydroxyethyl bromoisobutyrate into it. Immediately after that, add 170 µL (2.62 mmol) methane sulfonic acid (MSA) in this flask in air-free mode. Continue the stirring for 30 minutes set in a pre-heated oil bath at 40 °C. After 30 minutes, place the reaction mixture in an ice bath, wait to reach the temperature at 0 °C (check by thermometer) and add 380 µL (2.18 mmol) N, N-diisopropylethyl amine (DIEA) into the flask. Place the reaction flask in a stir plate and continue the reaction at room temperature (RT) by stirring for a week. DO NOT FORGET TO LABEL YOUR REACTION!!

****Safety:** MSA and DIEA may cause skin burns if touched. Wear protective gloves while handling these. Wash your hands with plenty of water for at least 15 minutes, in case there is any skin contact.

Handout 2

Laboratory 2: Polyalanine Purification

Note:

(1) All activity must be performed inside fume hood.

(2) Follow the waste disposal instructions properly for waste diethyl ether.

Purification procedure:

Each team will collect a 250 mL beaker and fill that with 100 mL diethyl ether. You should set up an ice bath and place the beaker filled with diethyl ether into it. Wait till the liquid is chill. Meanwhile stop your polymerization reaction and dilute the crude product with ~10 mL DMSO. Use a glass pipette and precipitate this crude mixture into the chilled diethyl ether twice. **Follow Instructor's direction**: PRECIPITATE CRUDE MIXTURE INTO DIETHYL ETHER DROP BY DROP. DO NOT ADD THE WHOLE MIXTURE ALL AT ONCE. A white solid product should appear, decant the top, collect the solid and wash it with 50 mL chilled diethyl ether twice. Collect the solid white product in an aluminium petri dish and dry it on a hot plate set at 40 °C for 30-40 minutes. Upon drying, store the product in a scintillation via, label it with your team number and keep in a vacuum oven set at 35 °C for a week for

additional drying. **Follow Instructor's direction**: HOW TO OPEARATE A VACUUM OVEN.
****Safety:** Diethyl ether is an eye irritant if touched, and harmful to the lung if inhaled. Wash hands and flash eyes with plenty of water for at least 15 minutes, in case there is any skin or eye contact with diethyl ether.

Handout 3

Laboratory 3: ESI-MS Sample Preparation and Introduction to CHEMDRAW Software

Note:

(1) Collect your product polyalanine from vacuum oven and take a photograph of your sample. Insert that in your formal lab report, which is due at the end of this course.
(2) Store it in assigned lab drawer for further use.
(3) Each team should prepare an ESI-MS sample with their individual product following the instructions below (Follow ESI-MS sample preparation method, described below). Sample should be handed over to the instructor. The instructor will give an overview of the ESI-MS instrument located on the university campus. The instructor will perform ESI-MS measurement (ESI-MS characterization and operation conditions are described below) and upload it to the CANVAS in the next 2-3 days.
(4) Download ChemDraw Prime software, find the download instructions below.
(5) After downloading, follow the instructions given by the instructor on how to draw a molecule and analyze its mass using this software.

ESI-MS Sample Preparation Method:

Each team should collect a 1 mL vial from instructor to prepare a mass spec sample. Prepare a solution in acetonitrile with the concentration of 1 mg/mL. PREPARE THE ESI-MS SAMPLE IN FUME HOOD. Vortex the solution to get a fully dissolved and clear solution. DONOT USE A STIR BAR TO AVOID CONTAMINATION. Label and hand over your sample to the instructor. **Safety:** Acetonitrile is a highly flammable liquid. Wash with plenty of water for at least 15 minutes, in case there is any skin contact.

ESI-MS Characterization:

High resolution (HR) mass spectral measurements for 3 samples will be performed using a Thermo Orbitrap mass spectrometer, which is connected to Thermo Vanquish UPLC system via an electrospray ionization (ESI) source as interface. Mobile phase is 50% Acetonitrile + 0.2% Formic acid (v/v) at 200ul/min for the direct injection. The conditions of MS analysis are as follows: the mass spectrometer parameters are positive ion mode, ion spray voltage at 4000 V, capillary voltage at 41V, capillary temperature at 275 °C, sheath gas flow rate at 40 psi and auxiliary gas flow rate at 20 psi. The scan spectra include m/z 400 to 2000. The instrument m/z values are calibrated using the manufacturer's ESI Positive Ion calibration mixture. All data analysis will be performed using Thermo Xcalibur™ software. Accurate mass measurements will be performed at high resolution (resolving power of 60,000 FWHM at m/z 400).

Download instruction of ChemDraw Prime software:

Use the following (Self-registration) details below to download the ChemDraw Prime software (Medium Academic Institutional License) to your computer or laptop.

1. Self-Registration
   Click on link: https://informatics.perkinelmer.com/sitesubscription/

You can self-download and self-activate this software following the directions in the Quick-Start-Guide (https://connect.revvitysignal.com/sitesubscription/pdf/ChemDraw_QUICK_START_Activation_Guide.pdf).
Use the Activation ID and Entitlement ID as posted in CANVAS announcement.

Handout 4

Laboratory 4: ESI-MS Data Analysis for Polyalanine

Note:

(1) Analyze your obtained ESI-MS data with regards to the polypeptide masses drawn by the ChemDraw software. Identify the +1, +2 peaks. Find out whether the peaks we obtained represent nominal, average or monoisotopic mass?
(2) Conclude how many repeat units of alanine are in your product. Justify your answer based on the acquired ESI-MS spectra and ChemDraw software drawn molecules' masses.
(3) Participate in the data discussion with your peers and the instructor.
(4) Insert your (i) labeled MS and (ii) ESI-MS sample preparation and (iii) ESI-MS operation conditions in the formal lab report, which is due at the end of this course. Attach a photograph of your polyalanine sample to the lab report.

Handout 5

Laboratory 5: Electron Microscopy, and Thermal Analysis of polyalanine. Introduction to Image J Software

Instructor Note:

(1) Each team should prepare a sample with their individual Polyalanine for TEM following the instructions below (Follow TEM sample preparation method). The instructor will give an overview of the TEM instrument located on the university campus. The student helper will perform TEM imaging (Instrument operation condition is described below). The instructor will upload the images to CANVAS in the next 2 days.
(2) Each team should also collect two 20 mL scintillation vials, and fill those with 20 mg of dried polyalanine powder. These two samples are for thermal analysis including TGA and DSC. Label those vials with your Team information. Samples should be handed over to the instructor. The instructor will discuss the theoretical details and operational condition of the TGA and DSC instruments in a separate lecture session. The instructor will perform these two measurements (operation conditions are described below) and will upload it to the CANVAS in the next 2 days.
(3) Download Image J software, find the download instructions below.
(3) After downloading, follow the instructions given by the instructor in the post-lab lecture of how to measure a spherical particle size using this software. Use an excel sheet, tabulate ~40-50 aggregate size, determine average and standard deviation. Report these values in your lab report.

TEM Sample Preparation Method:
Each team should collect a 20 mL scintillation vial from the instructor to prepare TEM samples. You should dissolve 10 mg polyalanine in 10 mL DMSO followed by 15 minutes vortex until a clear solution appears. Add 1 mL water into this solution dropwise. **Follow Instructor's direction**: ADD WATER DROP BY DROP TO DRIVE SELF-ASSEMBLY. DO NOT ADD ALL AT ONCE. Keep this mixture in an incubator shaker for 30 minutes, temperature set at 27 ºC and fan speed set at 50 rpm (USE THE SAME PARAMETERS SET IN **Figure S1**).

Meanwhile, prepare a dialysis bag filled up with the polyalanine self-assembled solution. Collect a Float-A-Lyzer G2 Dislysis bag from your instructor, fill up the bag with water using a dropper and immerse it in a beaker filled with water for soaking the outside too. After 10 minutes, lift the bag carefully from the water filled beaker. Replace the water of the dialysis bag with your self-assembly solution and float that in a 4L conical flask filled with water (**Follow Instructor's direction properly about setting up dialysis bag**). This 4L conical flask should be equipped with a large stir bar and sit on a mechanical stir plate. The solution should be dialyzed for 3 days. A DIALYSIS SETUP IS SHOWN IN **Figure S2**. During the dialysis, DMSO will be removed slowly, and the self-assembled nanostructures remain undisturbed. After 72 hours, one student assistant will stop the dialysis, collect the content from the bag and will take images by TEM.

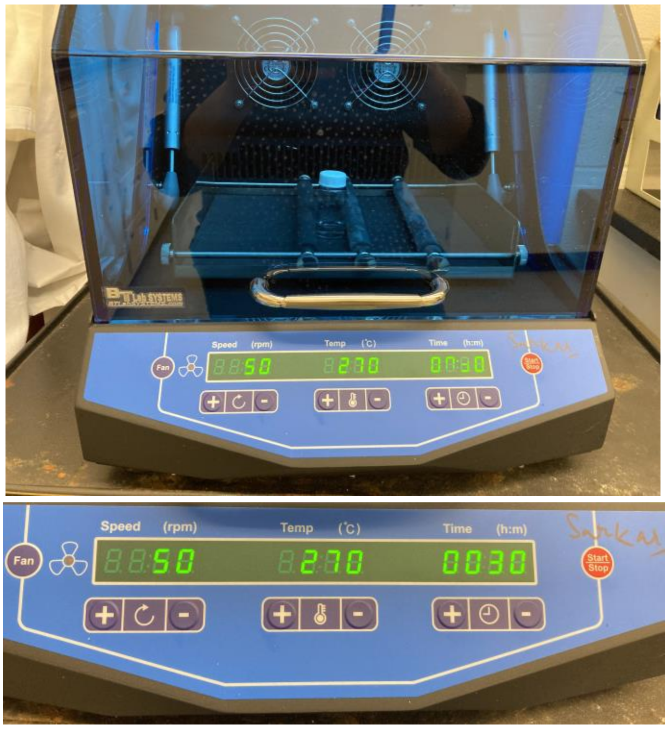

Figure S1. Polyalanine self-assembly sample in an incubator shaker. Set parameters are as Temperature: 27 ºC, Fan Speed: 50 rpm and Time: 30 minutes. Use these conditions when setting up your reaction.

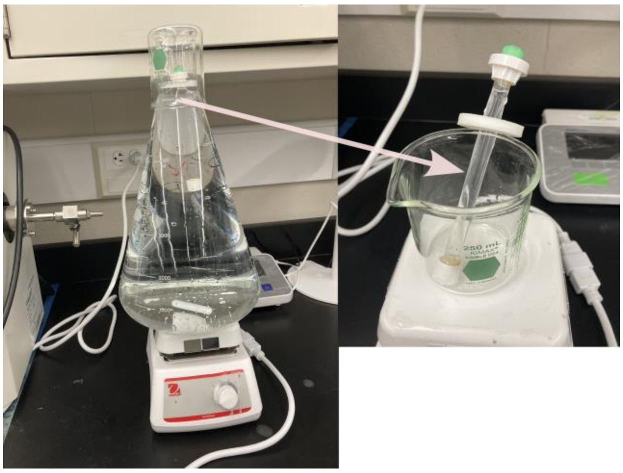

Figure S2. Dialysis setup.

TEM Characterization:

Transmission Electron Microscopy (TEM) images for polyalanine samples will be acquired using a Hitachi H-7500 tungsten/LaB6TEM operated at 120 kV. The resultant solution (one drop) will be placed on a carbon coated copper grid (Electron Microscopy Sciences, USA), and then placed on a piece of filter paper to remove excess solvent, and air dried before imaging. TEM samples will not be stained before imaging.

TGA and DSC Characterization:

Thermal analyses of the synthesized polymers will be performed with a Differential Scanning Calorimeter TA Instrument Discovery DSC in a temperature range of 25 to 300 ºC at a heating rate of 10 ºC min^{-1} under a nitrogen flow of 60 mL min^{-1}. The glass transition temperature (T_g) will be determined from the second heating trace and reported as the midpoint of the thermal transition. Thermal degradation of the polymers will be investigated by thermogravimetric analysis (TGA) performed with a TA Instruments Discovery TGA. Measurements will be conducted from 25 to 600 ºC at a rate of 10 ºC min^{-1} in a nitrogen flow of 60 mL min^{-1}.

Download instruction of Image J software:

Use the following link to download the Image J software to your computer or laptop.

Click on link: https://imagej.nih.gov/ij/download.html

You can self-download and self-activate this software following the directions in the link https://imagej.nih.gov/ij/docs/install/windows.html (Window Installation)
https://imagej.nih.gov/ij/docs/install/osx.html (Mac Installation)

Handout 6

Laboratory 6: TEM/TGA/DSC Data Analysis and Polystyrene Synthesis

Note:

(1) **TEM Data Analysis**:

(i) Download your corresponding TEM images from CANVAS. Follow the instructions regarding use of Image J discussed in the post-lab lecture on Week 5. Measure 40-50 spherical particle/aggregate size. Report average aggregate size along its standard deviation. Make a histogram with this size distribution.

(ii) Participate in the data discussion with your peers and the instructor.

(iii) Attach the TEM images along the histogram in the formal lab report, which is due at the end of this course.

(2) **TGA and DSC Data Analysis**:

(i) Download your corresponding TGA and DSC spreadsheets from CANVAS. Plot that as XY form. Determine decomposition temperature (T_d) from the TGA profile and melting temperature (T_M) from its DSC profile.

(ii) Explain what T_{d10} and $T_{d,50}$ is in a TGA profile.

(iii) Participate in the data discussion with your peers and the instructor.

(iv) Insert your TGA and DSC profile along conclusion in the formal lab report, which is due at the end of this course.

(3) **Polystyrene Synthesis via Activators Regenerated by Electron Transfer for Atom Transfer Radical Polymerization (ARGET ATRP)**

Note: Please read the article below. Write down the mole ratio of monomer to initiator to catalyst, ligand and reducing agent in your notebook, that is discussed in the pre-lab lecture. [HINT: reagent ratio of [monomer]:[initiator]:[L]:[Cu(I)]:[Reducing agent] = 150:1:0.105:0.005:0.1]. Experimental procedure described in this paper should be written in your notebook. Annotate any deviation or changes that you will do in this experimental procedure in this week's lab. Justify your changes or the effect of changes on the formal lab report, which is due at the end of this course.

Reference Literature:

Sarkar, A.; Stefik, M. Robust Porous Polymers Enabled by A Fast Trifluoroacetic Acid Etch with Improved Selectivity for Polylactide. *Mater. Chem. Front*. **2017**, *1*, 1526-1533.

Synthesis procedure:

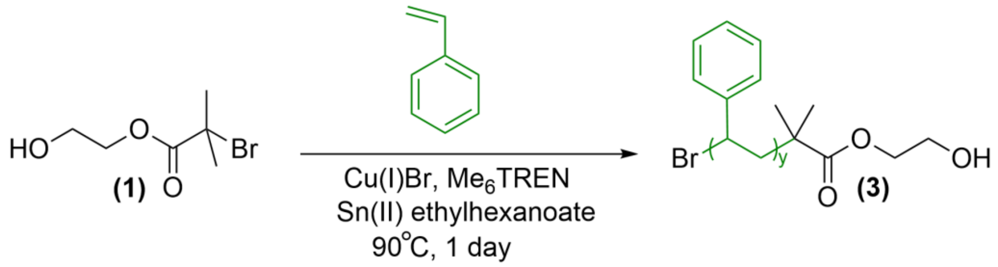

THIS EXPERIMENTAL SETUP IS SHOWN BELOW STEP-BY-STEP (**Figure S3**). FOLLOW THESE STEPS CAREFULLY AND CHECK YOUR EXPERIMENT SETUP WITH THE INSTRUCTOR. Each team should take one flame dried Schlenk flask equipped with a stir bar and go to the assigned hood space (**Figure S3a**). Prepare a neutral alumina column and pass 40-50 mL styrene monomer through it (REMINDER: ALL THE STEPS DESCRIBED HERE WILL BE PERFORMED IN YOUR ASSIGNED FUME HOOD) (**Figure S3b**). Collect 33 mL (290 mmol) column treated inhibitor free styrene monomer and pour that in the Schlenk flask carefully. DISCARD THE REMAINING STYRENE IN THE WASTE DISPOSAL CONTANER. Add 278 µL initiator (1.92 mmol) into it and attach a rubber septum into the flask. Seal the flask with a copper wire (**Figure S3c**), CHECK YOUR FLASK WITH THE INSTRUCTOR BEFORE PROCEEDING TO THE NEXT STEP. The flask will be purged with nitrogen gas for 15 minutes (**Follow Instructor's direction**: HOW TO ADJUST the PRESSURE ON A NITROGEN GAS CYLINDER REGULATOR) (**Figure S3d**). Separately, prepare a catalyst stock solution in a separate 5 mL vial. Prepare the stock solution (pale green color) of 1 mL Toluene containing 1.4 mg (0.0096 mmol) Cu(I)Br, 54 µL (0.202 mmol) Me_6TREN and 62 µL (0.192 mmol) Sn (II) ethyl hexanoate reducing agent (**Figure S3e**). Add this solution quickly through a syringe (FOLLOW THE INSTRUCTION GIVEN BY THE INSTRUCTOR) into the reaction flask under flowing nitrogen gas (**Figure S3f**). Place this reaction flask in a pre-heated oil bath set at 90 ºC (**Figure S3g**). Make sure the thermocouple is immersed in the oil bath fully (**Figure S3h**). Continue the reaction. The instructor and one student assistant will remove the reaction from heat after a day (27 hours) and store the reaction flask in the fridge to avoid side reactions or unwanted polymerization. DO NOT FORGET TO LABEL YOUR REACTION!!

Additional Note:
To demonstrate a visual change in reaction viscosity (**Figure S3c** (before polymerization) and **Figure S4b** (after polymerization)) and having enough polymer product after purification, longer reaction could be considered. However, instead of a day, the PS synthesis could be done in a shorter time. Please see GPC data of a PS synthesized in 16 hours, **Figure S14**.

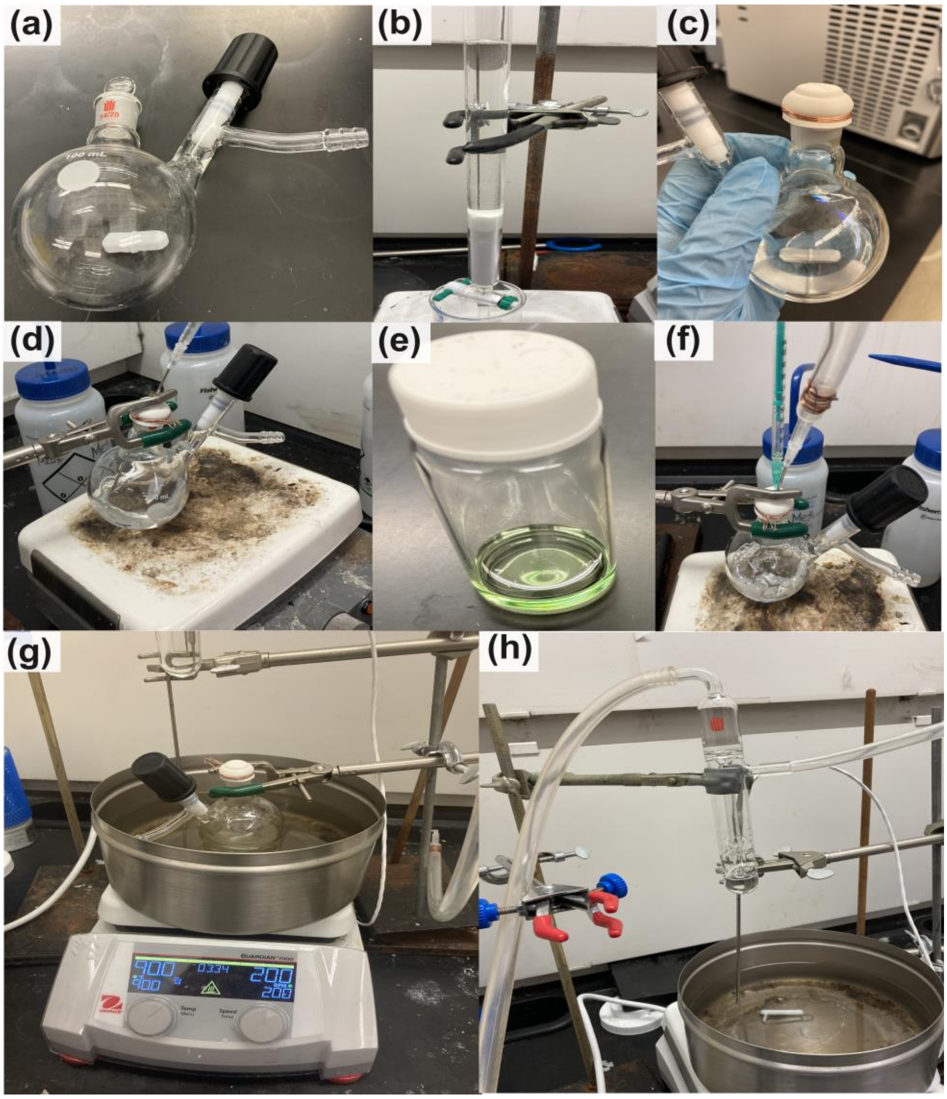

Figure S3. Polystyrene synthesis set up: step-by-step instruction.

****Safety:** (i) Each team should conduct all reaction steps described in **Figure S3** under the supervision of instructor.
(ii) All steps must be performed inside the hood. Alumina may cause lung damage, always use in a fume hood. Styrene is a highly flammable liquid and an eye irritant. Wash with plenty of water for at least 15 minutes in case there is any eye contact. Me_6TREN can cause severe skin and eye damage, wear protective goggles and gloves while handling this and wash hands and face thoroughly after using it. Use protective goggles and gloves (change frequently, if needed) while working with a high-temperature oil bath.
(iii) Students will start the polymerization; upon completion of the reaction, the polymerization flask will be removed from the heat by the instructor and one student assistant. Reaction flasks will be stored in fridge till the next lab period.

Handout 7

Laboratory 2: Polystyrene Purification and Samples Preparation for ^{1}HNMR, GPC, TGA and DSC.

Note:

(1) All activity must be performed inside fume hood.

(2) Follow the waste disposal instructions properly for waste methanol.

Purification procedure:

Each team will collect a 600 mL beaker and fill that with 300-400 mL methanol. You should set up an ice bath in the hood and place a beaker filled with methanol into it (**Figure S4a**). Wait until the liquid becomes chilled. Meanwhile grab your labeled polymerization flask from fridge, expose the solution to the air and dilute the crude product with ~10-20 mL tetrahydrofuran (THF). THE SOLUTION VISCOSITY SHOULD BE SOMETHING LIKE THAT COULD EASILY FLOW, CHECK YOUR SOLUTION VISCOISTY WITH THE INSTRUCTOR (**Figure S4b**). Use a glass pipette and precipitate this crude mixture into chilled methanol (**Figure S4c**). **Follow Instructor's direction**: PRECIPITATE CRUDE MIXTURE DROP BY DROP. DO NOT ADD THE WHOLE MIXTURE ALL AT ONCE. A white solid product should be deposited at the bottom, filter, collect the solid and wash it with 50 mL chilled methanol twice (**Figure S4d**). Dry the solid white product in the air for 10-15 minutes first, and then dry it in a vacuum oven pre-set at 65 ºC for an additional 30-40 minutes (**Figure S4e**). DO NOT FORGET TO LABEL YOUR PRODUCT BEFORE PLACING IN A VACUUM OVEN.

Safety: THF is a highly flammable liquid and may form explosive peroxide. THF may cause eye and respiratory tract irritation. Methanol is flammable and volatile, keep it away from hot plate or oil bath while working in a hood.

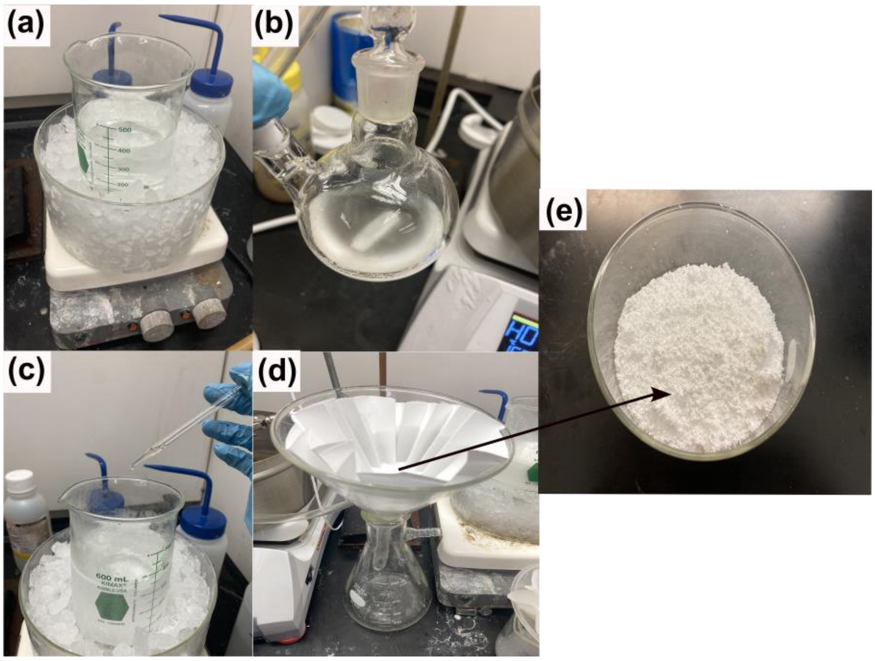

Figure S4. Polystyrene purification: step-by-step instruction.

****Safety:** All purification steps must be performed inside the hood. Liquid waste must be disposed of in a labeled and sealed container, as instruction given. Wear protective goggles and gloves throughout the whole experiment.

^{1}HNMR Sample Preparation Method:
Each team should collect a 10 mL vial from instructor to prepare ^{1}HNMR sample. PREPARE THE NMR SAMPLE IN FUME HOOD. Measure 10-15 mg dried polystyrene into it. Add 1 mL Deuterated chloroform ($CDCl_3$) into it using a 1 mL syringe. Vortex the solution for 1-2 minutes to get a clear solution. DONOT USE A STIR BAR TO AVOID CONTAMINATION. Transfer this solution (~0.5-0.6 mL) into an NMR tube using the same syringe. CAUTION: SYRINGE IS DANGEROUS. USE WITH CAUTION. At the end trash the syringe and needle both in syringe waste container (RED BOX, **Figure S5**) Label and handover the NMR tube to the student helper for NMR measurement. **Safety:** $CDCl_3$ is highly flammable and suspected of being a carcinogen. Work in a hood while using it.

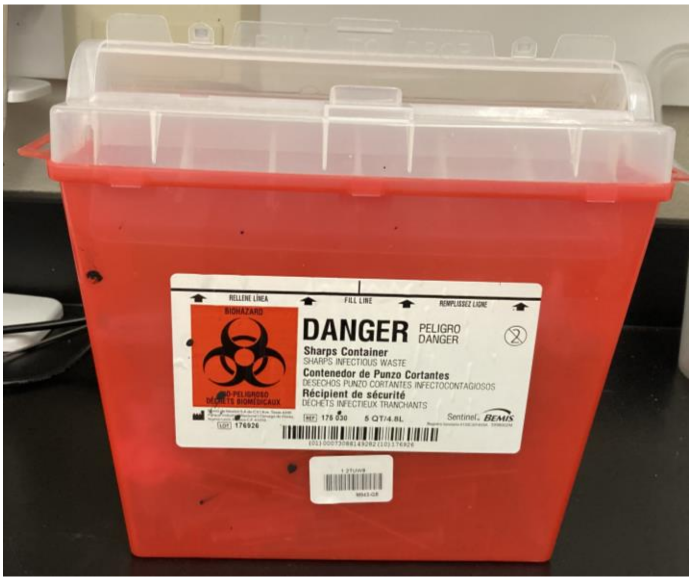

Figure S5. Syringe waste container.

GPC Sample Preparation Method:
Each team should collect a 10 mL vial from the instructor to prepare GPC sample. PREPARE THE GPC SAMPLE IN FUME HOOD. Measure 5 mg dried polystyrene into it. Add 5 mL tetrahydrofuran (THF) into it using a 10 mL syringe. Vortex the solution for 1-2 minutes to get a clear solution. DONOT USE A STIR BAR TO AVOID CONTAMINATION. Pass this solution through a 0.2 µ GPC filter. Collect ~1 mL from this filtered solution in a 1 mL GPC vial. Label and hand over your sample to the instructor. The instructor will discuss the theoretical details and operational condition of the GPC instruments in the pre-lab lecture session. The instructor will perform this measurement (operation conditions are described below) and will upload your data to the CANVAS in the next 2 days. **Safety:** THF is a highly flammable liquid and may form explosive peroxide. THF may cause eye and respiratory tract irritation.

TGA and DSC Sample Preparation Method:
Each team should also collect two 20 mL scintillation vials, and fill those with 10-20 mg of dried polystyrene powder. These two samples are for thermal analysis including TGA and DSC. Label those vials with your Team information. Samples should be handed over to the instructor.

The instructor will perform these two measurements (operation conditions are described in Handout 6) and will upload it to the CANVAS in the next 2 days.

^{1}HNMR Characterization

All NMR experiments will be performed at 400 MHz Bruker Avance III HD NMR spectrometer. All spectra will be recorded in $CDCl_3$. The number of scans will be set to 64. You will analyze your spectrum using master nova software with the help of instructor.

GPC Characterization.

Polymers number average and weight-average molecular weights and dispersity will be determined by a Waters Alliance e2695 gel permeation chromatography (GPC) system equipped with a 717 plus autosampler, a 1525 HPLC pump, and a 2414 refractive index (RI) detector. Columns of PLgel 5 µm guard, PLgel 10 µm MIXED-B and PLgel 5 µm MIXED-C will be set at temperature of 30 °C. The columns will be calibrated with narrow polystyrene (PS) standards in the molecular weight range of 580 to 371,100 Da. The measurements will be carried out with the sample concentrations of 1 mg/mL eluted by THF with a flow rate of 0.5 mL/min. Instrument operation and data analysis will be performed using Empower software.

Handout 8

Laboratory 8: Polystyrene ^{1}HNMR Data Analysis (^{1}H NMR End-Group Analysis).

Note:

Please read the two articles below. **Read Article 1** to understand the ARGET-ATRP mechanism and apply that into your particular reaction. Insert this mechanism into your formal lab report, which is due at the end of this course. **Read Article 2** to understand how to do end-group analysis in ^{1}H NMR spectroscopy. In addition to that the instructor will also provide a thorough lecture on how to conduct end-group analysis on your NMR spectrum.

Reference Literature:

Article 1. Jakubowski, W.; Denizli, B. K.; Gil, R. R.; Matyjaszewski, K. Polystyrene with Improved Chain-End Functionality and Higher Molecular Weight by ARGET ATRP. *Macromol. Chem. Phys*. **2008**, *209*, 32-39.

Article 2. Wackerly, J. W.; Dunne, J. F. Synthesis of Polystyrene and Molecular Weight Determination by ^{1}H NMR End-Group Analysis. *J. Chem. Educ*. **2017**, *94*, 1790-1793.

(1) Combining the above information, measure the number average molecular weight (M_n) and % chain -end functionality of your synthesized polystyrene.

(2) Participate in the data discussion with your peers and the instructor.

(3) Insert your (i) NMR spectrum with end-group analysis, (ii) NMR sample preparation and (iii) NMR operation conditions in the formal lab report, which is due at the end of this course. Attach a photograph of your polystyrene sample to the lab report.

Handout 9

Laboratory 9: Polystyrene GPC, TGA and DSC Data Analysis.

Note:

(1) **GPC Data Analysis**:

(i) Download your corresponding GPC result from CANVAS. Report M_n, M_w and Đ.
(ii) Download the excel datasheet and plot a XY plot with retention time in minutes in X and detector response in Y axis.
(iii) Identify which detector and calibrations are used in this measurement?
(iv) Make a comment on your ARGET-ATRP reaction efficacy based on the gathered information from GPC.
(v) Participate in the data discussion with your peers and the instructor.
(iii) Attach the results in the formal lab report, which is due at the end of this course.

(2) **TGA and DSC Data Analysis**:
(i) Download your corresponding TGA and DSC spreadsheets from CANVAS. Plot that as XY form. Determine decomposition temperature (T_d) from the TGA profile and glass transition temperature (T_g) from its DSC profile.
(ii) Explain what T_g value indicates.
(iii) Participate in the data discussion with your peers and the instructor.
(iv) Insert your TGA and DSC profile along conclusion in the formal lab report, which is due at the end of this course.

Handout 10

Laboratory 10: Submission of a Formal Lab Report.

Note:

Please submit the lab report in the below format. Your lab report will be evaluated based on the quality and comprehensiveness of the objective, your observation, quality of the work you have done and comprehensiveness of the conclusions. Also, the style, grammar and clarity of scientific wording will be evaluated.

Section 3
Lab report Guideline

(1) Although you'll work in a group, your lab report should not be replica of each other's sentences/words. Please remember that copying each other's lab report is under academic dishonesty. And you'll get "0" for doing that.
(2) Use Microsoft Word to write your lab report, use "Times New Roman, font size 12, double spaced and 1" margin.

Lab report Template:
Title: Write a concise title **(5 points)**
Author Information: Name and address (experiments) was performed **(5 points)**
Abstract: Why do you perform these experiments? What was your primary goal? What is the significant outcome? **(10 points)**
Introduction: Description of topic/adopted synthetic pathway background, problems and hypothesis tested. **(10 points)**
Experimental Methods: Discuss what experiments/analysis you have performed each week. Add every detail like sample preparation condition, instrument operation conditions, etc. **(10 points)**
Results and discussion: Discuss all the results/skills you obtained/learnt in this lab. Add all analyzed data including ESI-MS interpretation (with structures and molar masses drawn by Chem Draw software), ^{1}H NMR end-group analysis, M_n/M_w/Đ of polymer based on the GPC, TEM image with average spherical aggregate size, TGA/DSC plots, etc. Insert photographs that you have taken during setting up your reaction. **(30 points)**

Hazards: Write 5 safety steps that you followed during these lab activities. Make sure these 5 points are relevant to your lab work, do not write the general safety protocol, for example "I wear safety goggles all the time". (**10 points**)
Conclusions: Your remark about the overall lab activity. Here you could mention any difficulties/challenges you faced during performing these lab activities, or any suggestions to improve that. **(10 points)**
Reference: Cite all the journals/literature that we discussed throughout the lab (properly formatted adhering to the ACS style guide). **(10 points)**

Section 4
Students' Data and Students' Performances

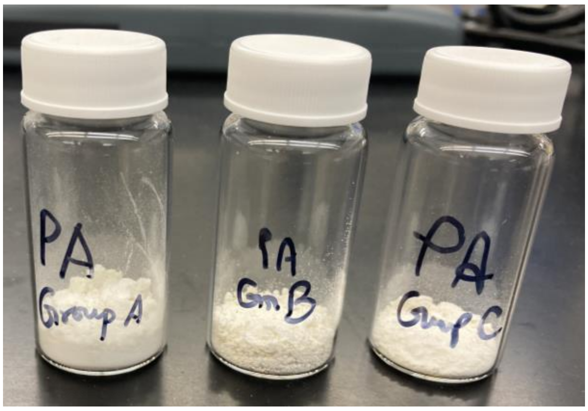

Figure S6. Polyalanine (PA) synthesized by 3 student teams.

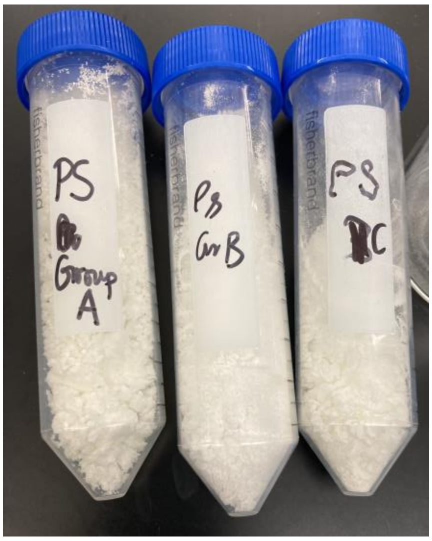

Figure S7. Polystyrene (PS) synthesized by student teams.

Polystyrene chain-end functionality (livingness) and molecular weight (M_n) determination by NMR end-group analysis:

Students synthesized polystyrene samples dissolved in $CDCl_3$ were run in a 400 MHz Bruker NMR instrument by a student assistant. The spectra were distributed in the class to the students and instructed to perform the end-group analysis. Students identified broad peaks responsible for aromatic (assigned by **d** and **d'**), and aliphatic regions (assigned by **b** and **c**) of the repeating monomer unit of polystyrene (**Figure S9**). They also identified the signal of initiator methylene proton (-CH_2, assigned by **e** and **e'**) at 3.50-3.80 ppm. Finally, the signal at 4.35-4.65 ppm was assigned to the proton **(a)** on the carbon adjacent to bromine of the growing polystyrene chain. In addition to that, students also identified the methyl protons of the initiator at 0.91-0.97 ppm (assigned by **f**). Comparing the integration of **a** peak to (**e** + **e'**) leads into % chain end functionality, following equation 1. For example, one student team obtained the % chain end functionality as 96% applying equation 1 and using the integration values shown in **Figure S8.**

$$\%\,Chain\ end\ functionality = 100\left[\frac{Ha}{\frac{e+e'}{4}}\right] \ldots\ldots\ldots\ldots(1)$$

$$\%\,Chain\ end\ functionality = 100\left[\frac{0.24}{\frac{1}{4}}\right]$$

$$\%\,Chain\ end\ functionality = 96\%$$

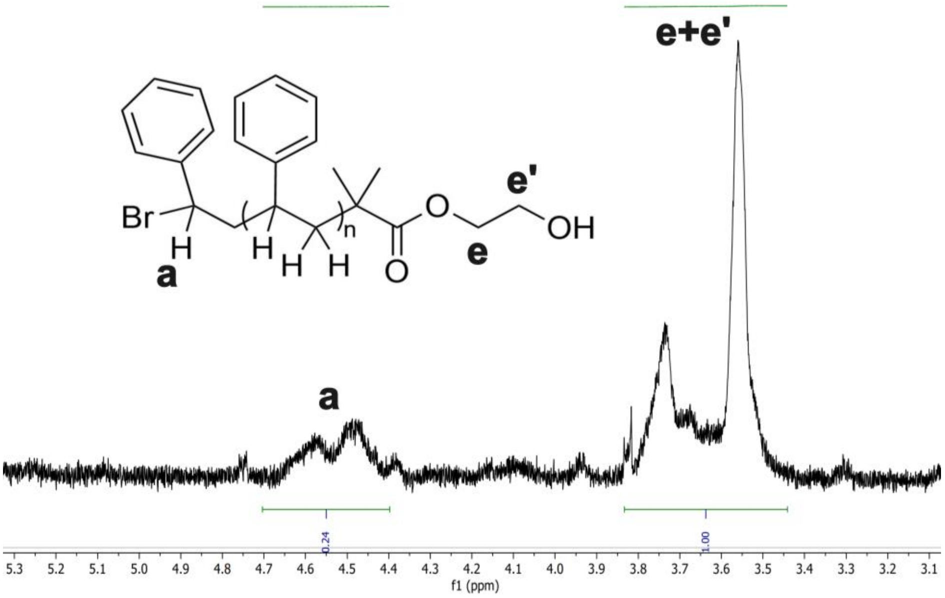

Figure S8. ^{1}HNMR spectrum for polystyrene used in determining % end-group functionality.

Next, students adjusted the overall integration values based on the end-group of the initiator, such as the integration for proton signal at 3.50-3.80 was normalized to 4 and multiplied all repeat unit proton signals by that value, integration values are shown in **Figure S9**.

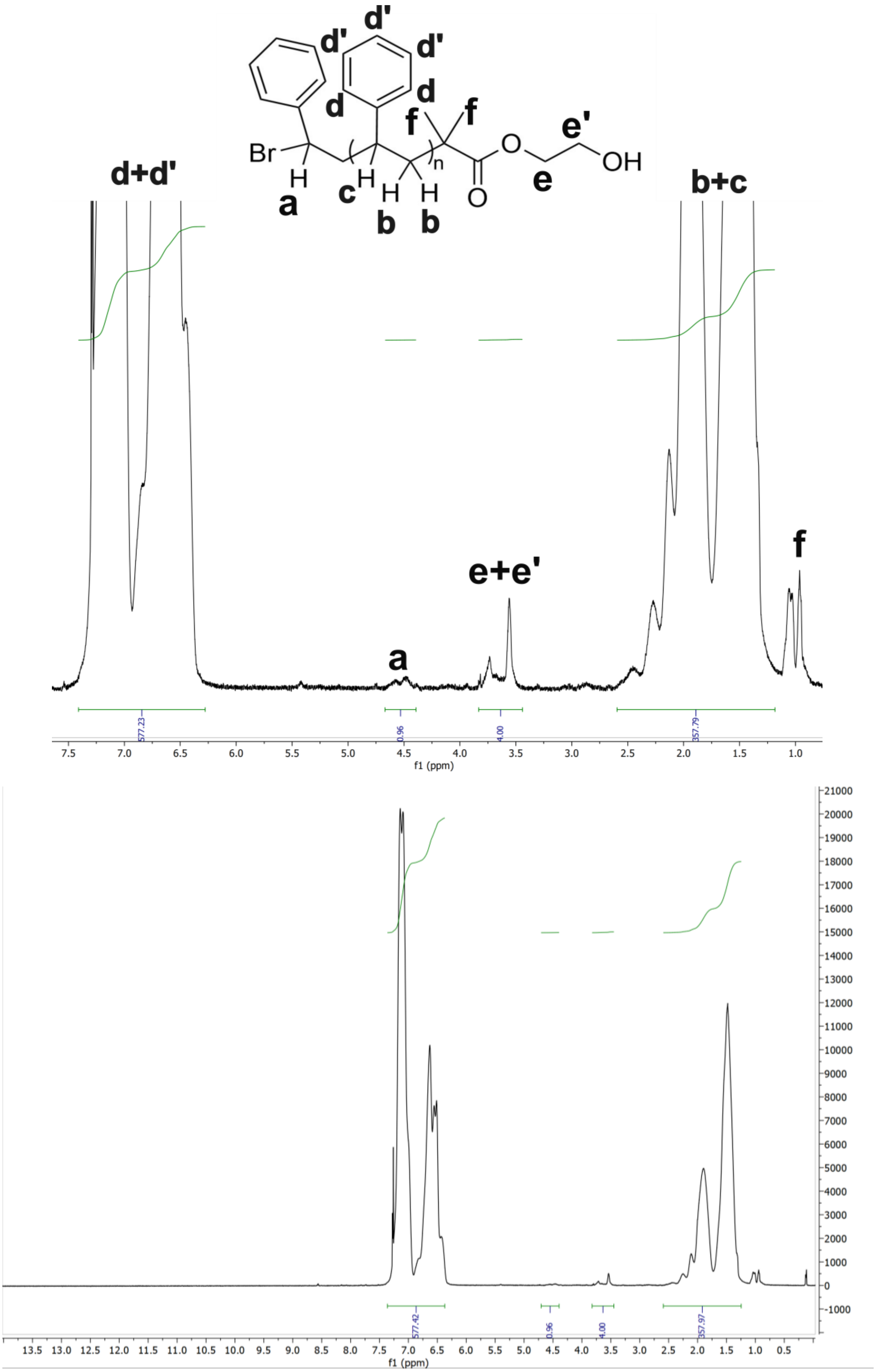

Figure S9. M_n estimated from the ^{1}HNMR spectrum for polystyrene (expanded view, top). Full spectrum (δ = 0-14 ppm) of the same is shown (bottom).

Using the **Figure S9**, students calculated the degree of polymerization (n) as below;

$(\text{Polystyrene})_n$ (considering 5 aromatic polystyrene protons, d+d') = (577.2/5) = 115.4

Or

$(\text{Polystyrene})_n$ (considering 3 aliphatic protons in backbone, b+c) = (357.8/3) = 119.2
Students relied on the value obtained from aliphatic region (b+c) as it was relatively cleaner area and not overlapped with other interfering proton signals compared to aromatic region that is overlapped with $CDCl_3$. Once n was set to be 119, this value was used to determine M_n using the equation 2 and molar mass of styrene as 104.14 $gmol^{-1}$.

$$Mn = n.monomer\ molar\ mass \ldots\ldots\ldots\ldots\ldots\ldots(2)$$
$$Mn = (119).(104.14)gmol - 1 = 12{,}392.7\ gmol - 1$$

A student team obtained the M_n as 12,392.7 $gmol^{-1}$ using **Figure S9** and applying equation 2.

Table S1. Participating students' performance average.

Tasks	Performance
**Activity #1**	All three teams (10 students, 100%) synthesized PA by NCA ROP successfully and analyzed the products' ESI-MS satisfactorily.
**Activity #2**	All three teams (10 students, 100%) synthesized PS successfully via ARGET ATRP and determined Mn and % chain-end functionality by 1HNMR end-group analysis. All three teams' synthesized PS was monomodal (confirmed by GPC, Đ<1.1), however, one team's (3 students, 30%) synthesized PS was found with broader dispersity (Đ>1.3).
**Activity #3**	All three teams (10 students, 100%) evaluated TEM images with correct estimation of self-assembled PA aggregates size. They also determined correct T_g and T_d for PS and PA from their corresponding DSC and TGA profiles.

Section 5. Exams

Take Home Exam 1 (CHEM 490), 50 points

***Your independent work/effort is <u>**MUST**</u> to get full points**

1. Show the free radical polymerization steps with an initiator AIBN and a monomer butyl acrylate. **[10 points]**

2. On the basis of your assessment of whether this polymerization would ideally proceed as a chain-growth polymerization with or without control, draw the expected profile for DP vs. % monomer conversion on the axes. [**5 points**]

3. We discussed in class that during polystyrene synthesis (via ATRP) side reactions can occur. Describe (with reaction equations) what unwanted side reactions can occur during polystyrene synthesis and how can it be prevented? **[10 points]**

4. During Laboratory Activity 1 you started the synthesis of Br terminated polyalanine in flame-dried glassware. Your instructor mentioned that the use of dry glassware or dry reagent/solvent are important. Why is that? Refer to the reaction scheme in Lab 1 Handout and your product ESI-MS data to answer this problem. **[5 points]**

5. Your instructor provided you with a polymer sample that has an average molecular weight of 100,000 g/mol and a dispersity (Đ) of 5. Determine the weight average molecular weight of this polymer sample. **[5 points]**

6. Draw a polymer structure as a copolymerization between styrene and hexyl acrylate monomers in presence of AIBN initiator. **[5 points]**

7. Write the general schematic reaction of ATRP and ARGET-ATRP. [**10 points**]

Take Home Exam 2 (CHEM 490), 50 points

*****Your independent work/effort is **MUST** to get full points

1. A newly synthesized polymer was run on the Small Angle X-ray Scattering (SAXS) machine, and the diffraction peak position found at θ = 10.2º. The wavelength of X-ray beam in the SAXS machine is set at 0.154 nm. Calculate domain (d) spacing in Å for that polymer. **[10 points]**
2. Discuss briefly the Flory-Huggins theory for homopolymer dissolution and diblock copolymer mixing. Label the cylinder (HEX) morphology in a typical AB deblock copolymer phase diagram [**10 points**]
3. A polymer sample is heated at 10 ºC/minute in air in a Thermogravimetry (TG). In this condition 20% degradation of this polymer was found at 200 ºC. On the contrary when this polymer was heated at 10º C/minute in nitrogen gas in the TG, the 20% decomposition was found at 350 ºC. Draw A TGA profile using this information. [**10 points**]
4. Draw the polymer formed by the step-growth polymerization of the following molecules. [**5X2 =10 points**]

a)

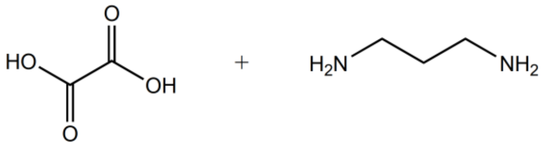

b)

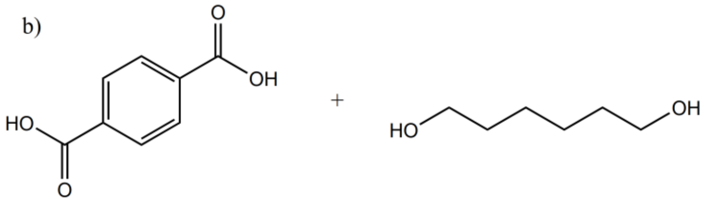

5. Write True/False for below sentences. [**2.5x4 =10 points**]

(a) Scattering profile and Electron Microscopy Images both data are required to confirm a nanoscale morphology.

(b) Dispersity 2 is quite common for step-growth polymerization whereas the dispersity must be <1.2 for controlled living polymerization.

(c) During the micelle sample preparation for TEM, your lab partner added 5 mL of water all at once instead of drop-by-drop. You think that would not affect your self-assembly.

(d) Schlenk flask is typically used to perform air-free synthesis.

Answer key

Take Home Exam 1

(1) Initiation

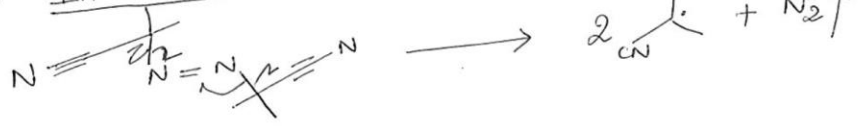

AIBN

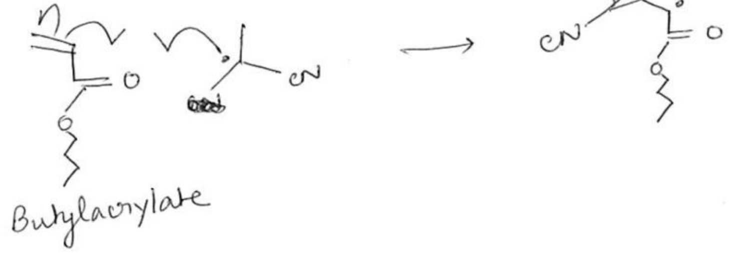

Butylacrylate

(2) Propagation

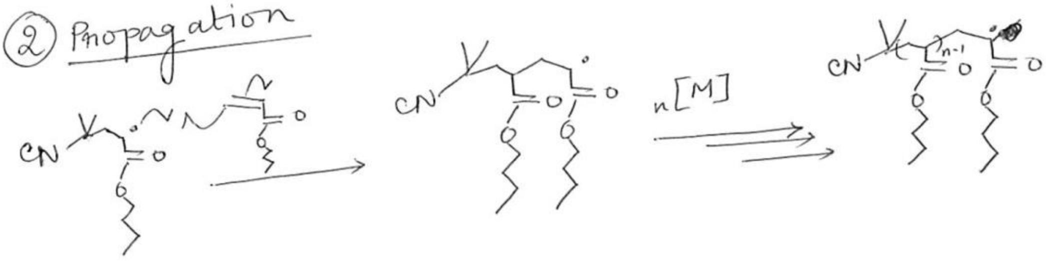

(3) Termination

(i) Coupling

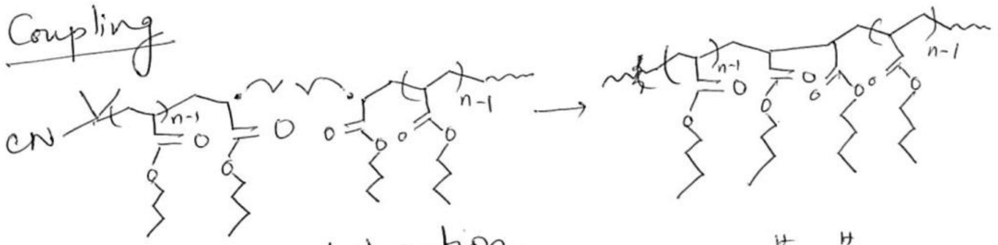

(ii) Hydrogen atom abstraction

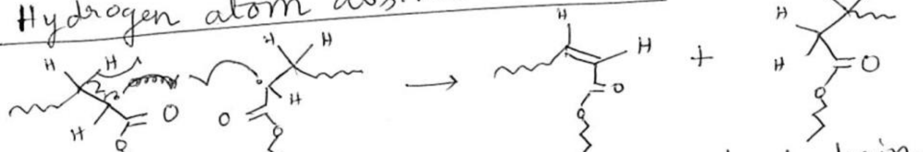

dead chain

2.

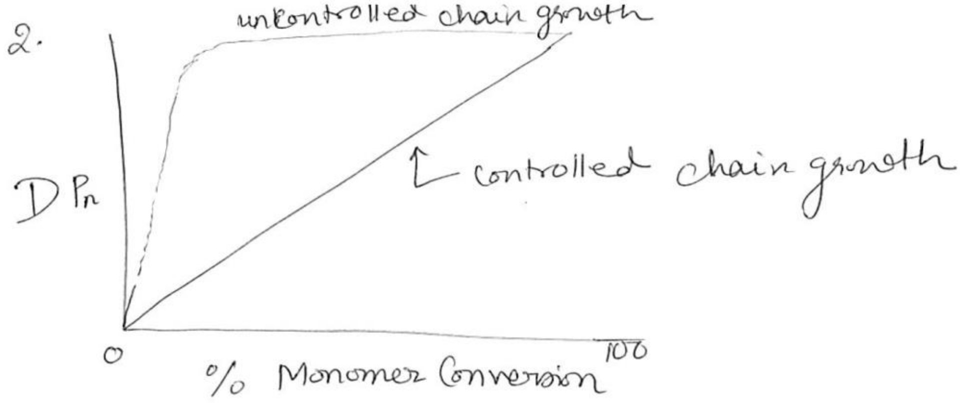

3. The chain end functionality of polystyrene during ATRP can be lost due to termination reactions & side reactions between growing polystyrene radical & Cu catalyst. Side reactions can happen via two ways, shown below.

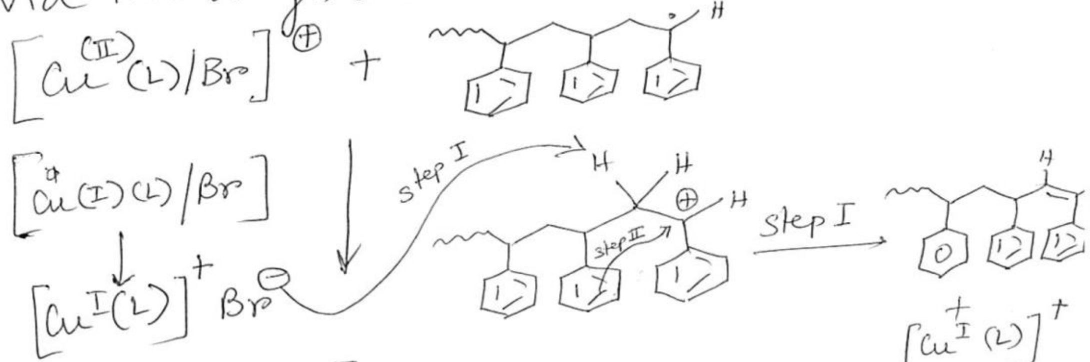

In both cases, HBr is eliminated when Cu(II)Br_2 reacts with propagating PS radicals & yields unsaturated or cyclic chain end.

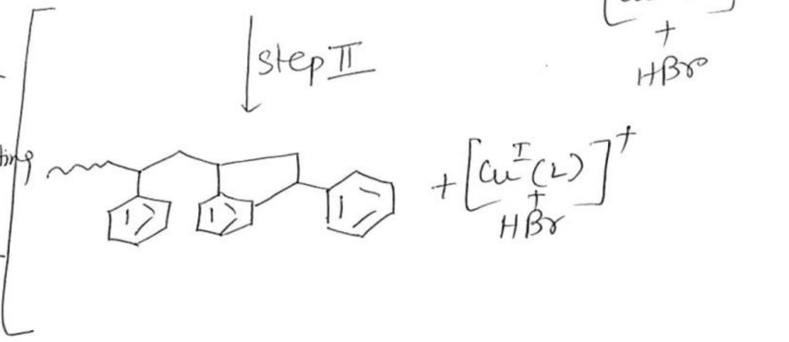

Besides these two, usual radical coupling & transfer can happen, shown below.

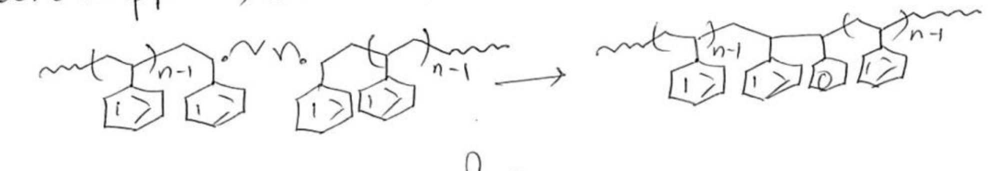

&

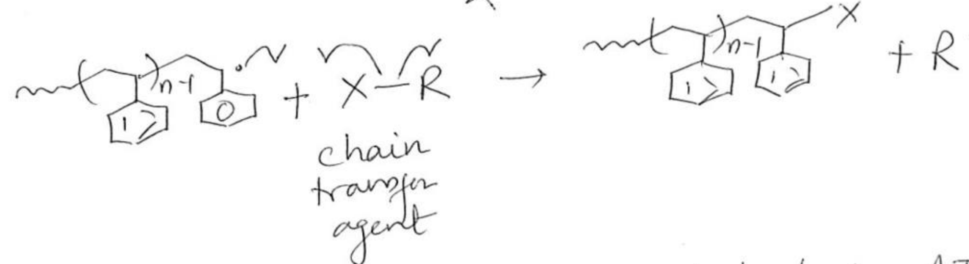

By decreasing amount of Cu catalyst in ATRP or using ARGET ATRP the catalyst induced side reactions can be prevented.

(4) Trace presence of water (in glass ware or moist reagents) may initiate alanine N carboxyanhydride predominantly instead of the initiator 2-hydroxy ethyl-2-bromoisobutyrate. Thus, polyalanine with desired end group may not be obtained. For an example, we aimed to have the polyalanine with the structure I. But, the MS analysis shows that our possible product structure is as II.

Structure I

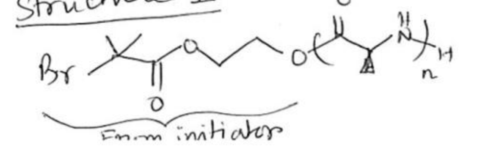

Structure II

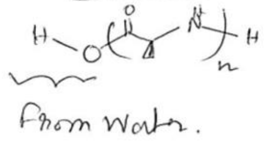

(5) $M_n = 100{,}000\ g\,mol^{-1}$ $Đ = 5$ $M_w = ?$

$Đ = M_w/M_n$ ∴ $M_w = Đ \times M_n = 500{,}000\ g\,mol^{-1}$

(6) 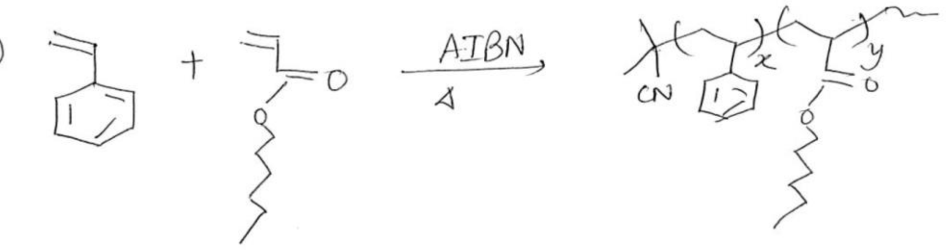

(7) ATRP

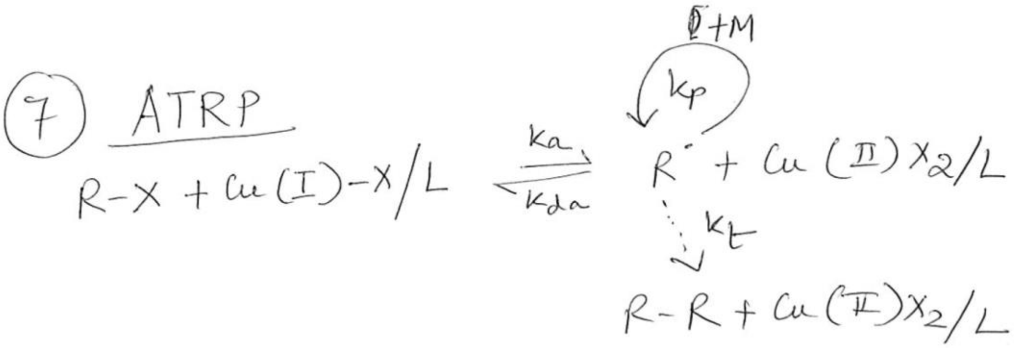

ARGET ATRP

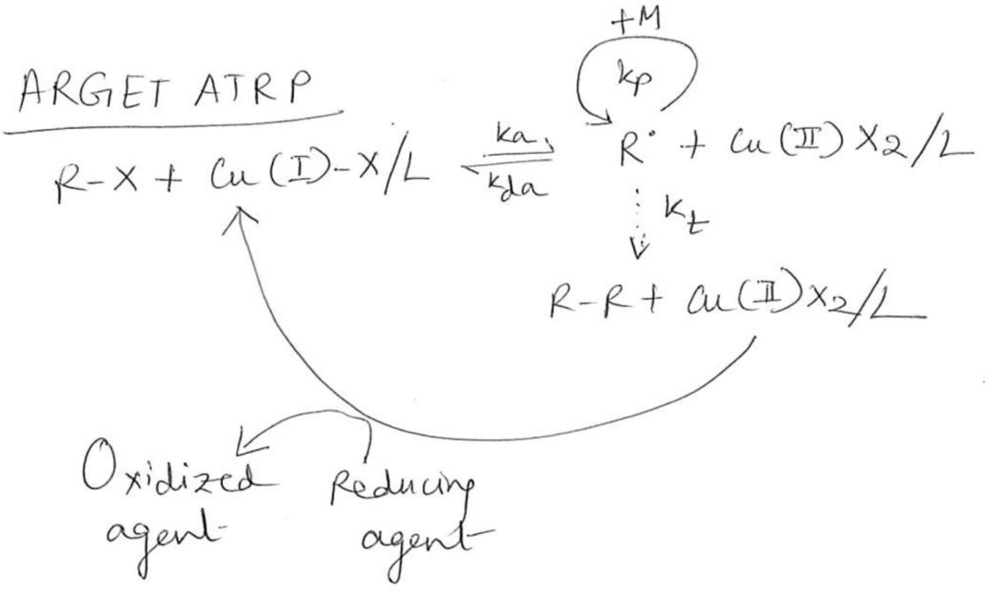

Take Home Exam 2 (Answer)

1. $d = \frac{2\pi}{q}$

$q = 4\pi\left(\sin \theta/\lambda\right) = 4\pi\left(\frac{\sin 10.2}{0.154\,nm}\right) = 14.4\,nm^{-1}$

$d = 2\pi / 14.4\,nm^{-1} = 0.436\,nm = 4.36\,Å$

2. $\Delta G_m = \Delta H_m - T\Delta S_m$ [For spontaneous dissolution]

Dissolution Entropy;

$\Delta S_{mixing} = \Delta S_{mixed} - \Delta S_{polymer} - \Delta S_{solvent}$

$\Delta S_{mixing} = -N_1 \ln(f_1) - N_2 \ln(f_2)$

or $\frac{\Delta S_{mixing}}{N} = -\frac{1}{x_1}\ln(f_1) - f_2 \ln(f_2)$

[N_1 = No. of solute molecule
N_2 = No. of polymer molecule]

Mixing produces positive entropy that scales with N

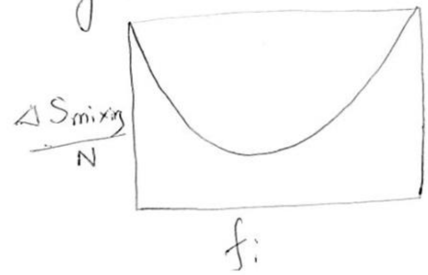

Entropy change is largest for nearly pure solution.

Dissolution Enthalpy;

Energy increment per monomer-solvent contact.

$\Delta \omega = \omega_{12} - \frac{1}{2}(W_{11} + W_{22})$

$\therefore$ Total number of contacts $= x N_2 Z f_1 = N_1 f_2 Z$

$\therefore \Delta H = (N_2 f_1 Z)\Delta \omega$

[Here ω is molecular interaction
ω_{11} = Solvent-solvent interaction
ω_{22} = Monomer-monomer "
ω_{12} = Monomer-Solvent ", Z = coordinate Number

$$\Delta G_{mix} = (N_2 f_1 Z)\Delta\omega + k_b T \left(N_1(\ln f_1)\right) + N_2(\ln(f_2)))$$

Polymer-solvent interaction parameter,

$$\chi_{12} = \frac{Z\,\Delta\omega}{\gamma} \quad [\gamma = kT,\ k = \text{constant},\ T = \text{Temperature}]$$

$$\Delta G_{mix} = \gamma\left(N_2 f_1 \chi + N_1(\ln(f_1)) + N_2(\ln(f_2))\right)$$

Complete free energy of mixing

$$\frac{\Delta G_{mixing}}{\gamma} = N_1 \ln(f) + N_2 \ln(1-f) + N_2 f \chi$$

$$\frac{\Delta G_{mixing}}{\gamma N} = \frac{N_1}{x_1 N_1 + N_2}\ln(f) + \frac{N_2}{x_1 N_1 + N_2}\ln(1-f) + \frac{N_2}{x_1 N_1 + N_2} f \chi$$

[x_1, x_2 & χ all are different variables]

For Homopolymer dissolution, the Flory-Huggins ~~interaction~~ solution theory will be

$$\frac{\Delta G_{mixing}}{\gamma N} = \frac{1}{x_1} f \ln(f) + (1-f)\ln(1-f) + (1-f) f \chi$$

For Diblock copolymer mixing, the Flory-Huggins theory will be

$$\frac{\Delta G_{mixing}}{\gamma N} = \frac{1}{x_1} f_1 (\ln f_1) + \frac{1}{x_2} f_2 \ln(f_2) + f_1 f_2 \chi$$

$x_1 + x_2$ = Chain length / degree of polymerization

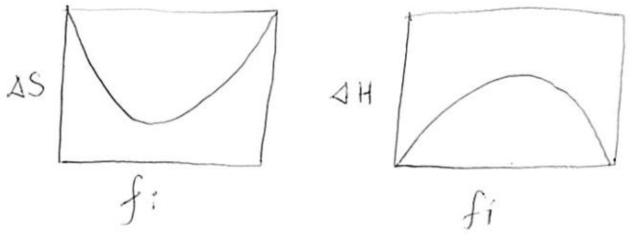

Transition at $\Delta G = 0$

$$\chi (x_1 + x_2) = \frac{1}{f(1-f)} \ln(f) - \frac{1}{f(1-f)} \ln(1-f)$$

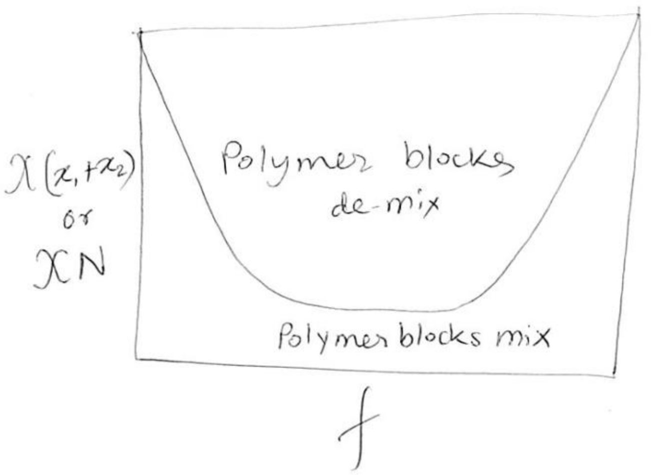

For AB Diblock copolymer

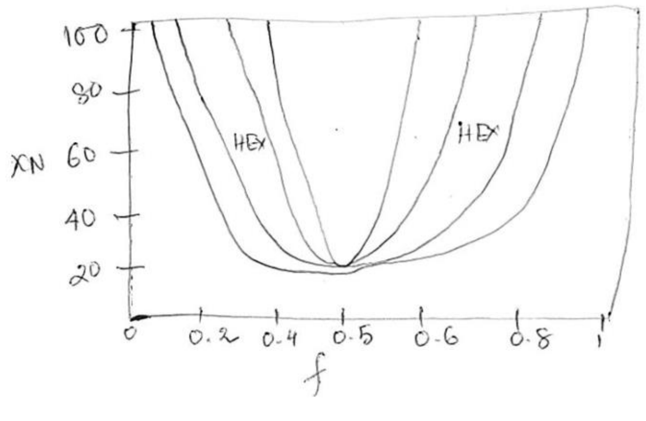

3

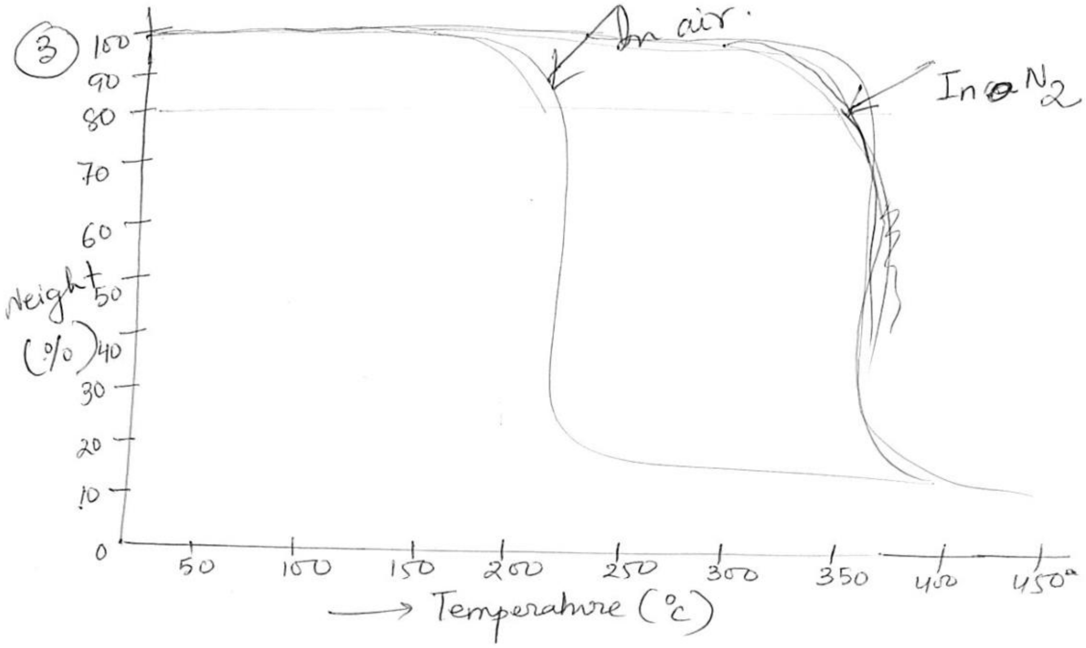

4 (a)

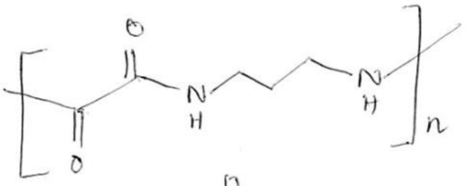

(b)

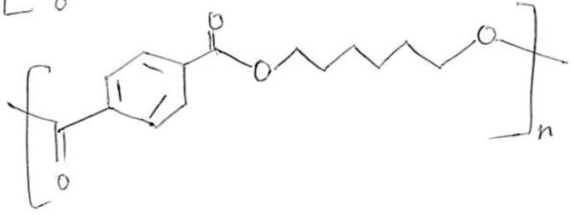

5. (a) True

(b) True

(c) False

(d) True

Section 6

Grading Rubric

Overall grade 350 point is divided into 4 major activities, please see it below:

1. students' performance in the lab during each week lab activity (100 points)
2. One formal laboratory report submission (100 points)
3. Two Take-home exams (50 points each)
4. an oral presentation (50 points)

Section 7
Additional Note

Additional Characterization Technique (FTIR)

Along with the NMR and GPC, another characterization technique Fourier Transform Infra-red (FTIR) for polystyrene could be added in this laboratory module in future. A representative FTIR spectrum along its summary peaks for the initiator 2-hydroxyethyl-2-bromoisobutyrate is shown in **Figure S10**, **Table S2**. Likewise, the spectra for styrene monomer and polystyrene along its detailed IR analysis is presented in **Figure S11**.

FTIR characterization: FTIR spectra of the initiator, monomer and polymer were carried out in a PerkinElmer Spectrum Two FT-IR spectrometer using attenuated total reflectance (ATR). Measurements were carried out in the range of 4000-650 cm^{-1} with a scan number of 32 and resolution of 8 cm^{-1}.

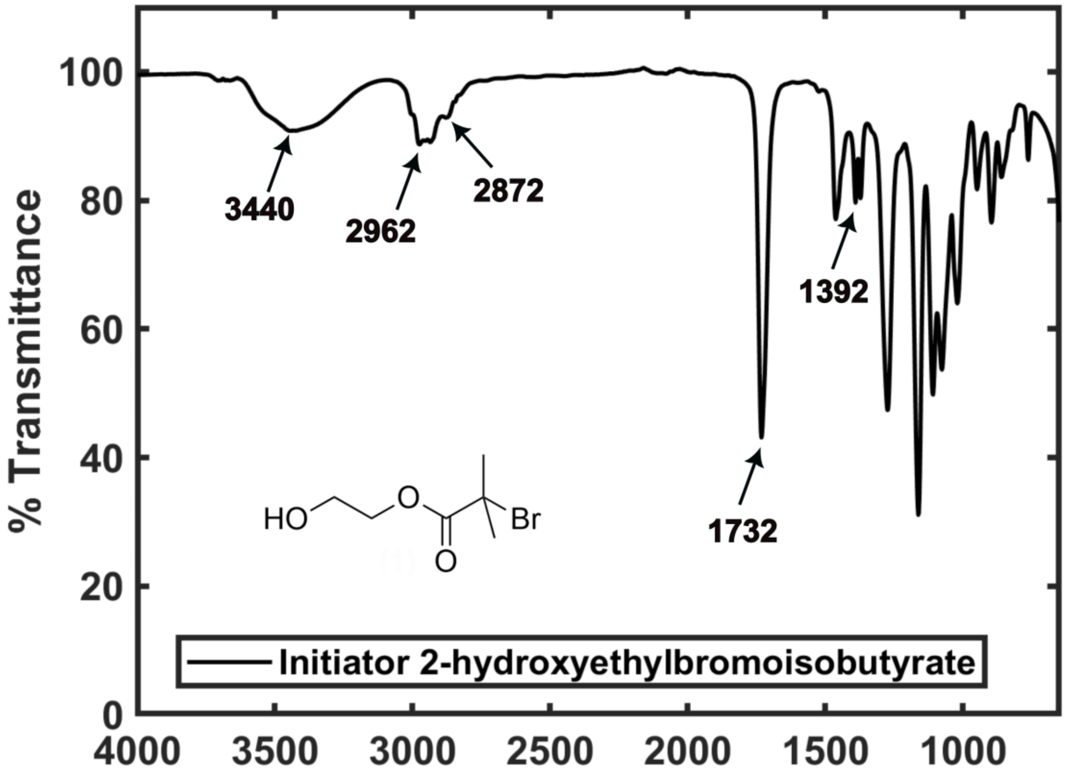

Figure S10. FTIR analysis of initiator 2-hydroxyethylbromoisobutyrate. Absorption bands are summarized in **Table S2**.

Table S2. Peak summary for initiator 2-hydroxyethyl-2-bromoisobutyrate.

**Peaks/absorption at (cm^{-1})**	**Stretching vibration responsible for**
3440	hydroxyl (OH) vibration.
2962, 2872	C-H vibration of -CH_2-CH_2-group
1732	ester C=O stretching vibration
1392	C-H stretching vibration of -CH_3-group

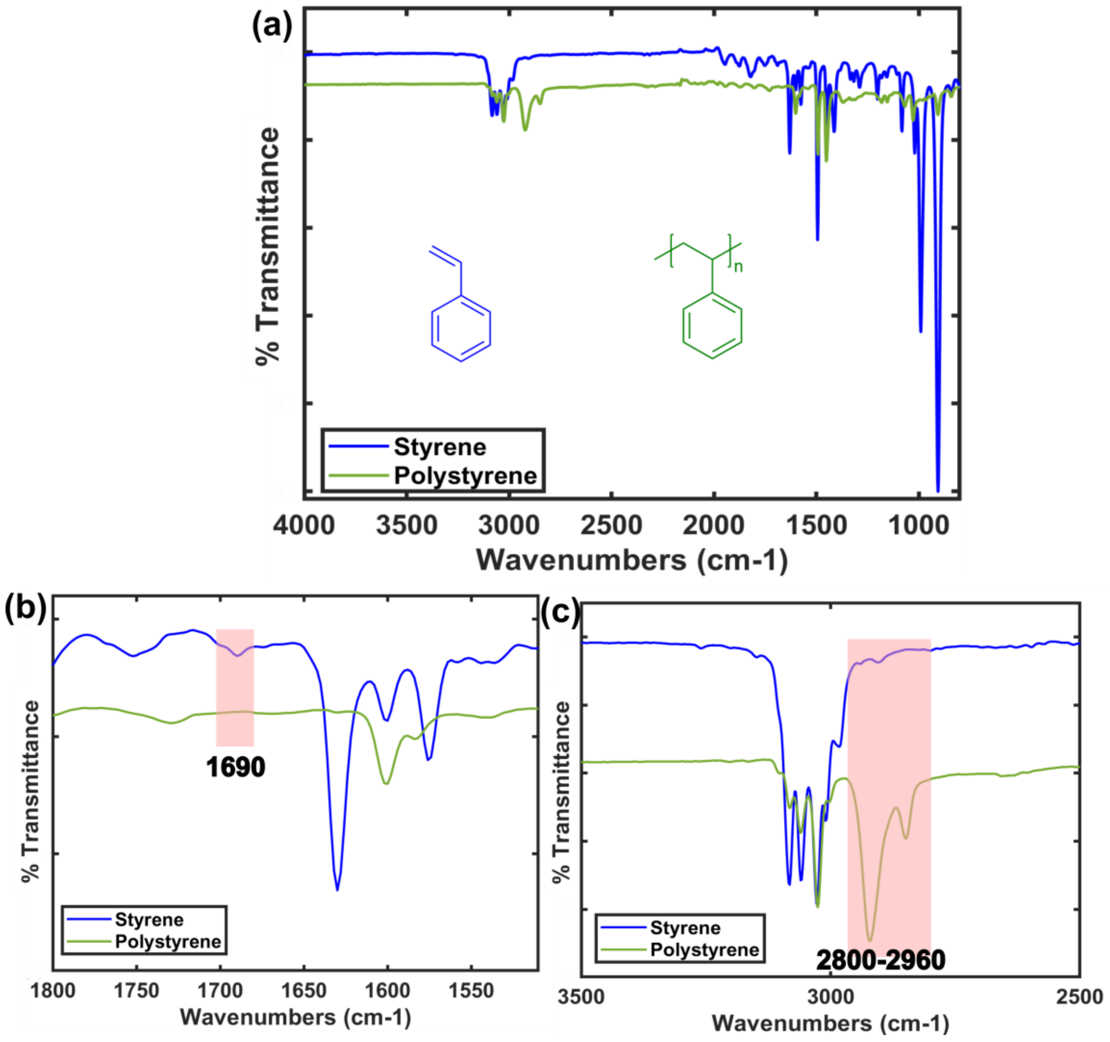

Figure S11. FTIR analysis of monomer styrene and polystyrene. Full spectra are shown in (a), and the expanded spectra are shown in (b-c).

FTIR spectra for PS (green) shows disappearance of signal at 1690 cm^{-1} (found in styrene monomer spectrum, blue) that corresponds to the C=C stretching vibration of vinyl group in styrene monomer (**Figure S11 b**). Additionally, strong peaks in the range of 2960-2800 cm^{-1} for PS are found that correspond to the C-H bond stretching which originates due to the polymer backbone formation (sp^3 carbon) (**Figure S11c**).

Polystyrene Synthesis (Alternative Low-cost Synthesis Approach)

The high costs of reagents often prohibit many institutions with certain budgets from training students in synthesizing organic molecules or polymers. Even though our reported synthesis approach for PS is successful, an alternative approach is presented here incorporating low-cost ligand and reducing agents. Instead of the Me_6TEN, relatively cheaper ligand N,N,N,N,N-pentamethyldiethylenetriamine (PMDETA) could be used. Likewise, to avoid the students' handling of Sn(II) ethylhexanoate, a milder and cheaper reducing agent ascorbic acid could be used. In addition, the synthesis could be conducted for a shorter period. Synthesis procedure and purifications are same as described in **Figure S3** and **Figure S4.** A few key modifications/changes could be applied in the procedure, described below.

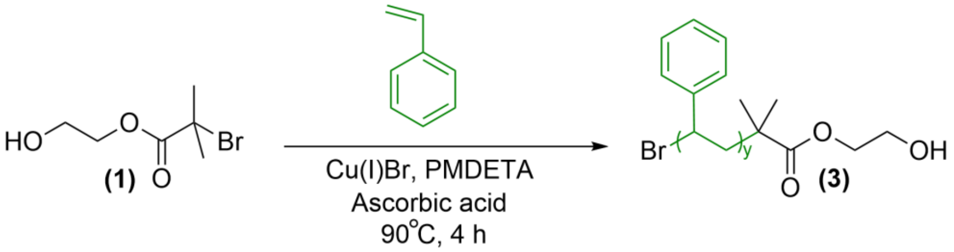

Synthesis and purification procedures:

Synthesis and purification procedures are the same as described in **Figure S3** and **Figure S4.** Briefly stated, the reagent ratio of [monomer]:[initiator]:[L]:[Cu(I)]:[Reducing agent] = 150:1:0.105:0.005:0.1 was used here. A mixture of 11 mL alumina column treated inhibitor free styrene monomer and 93 µL initiator was purged with nitrogen gas for 15 minutes. Separately, a catalyst stock solution of 1 mL THF containing $4x10^{-7}$ g Cu(I)Br, 14 µL PMDETA and 11 mg ascorbic acid were added into polymerization medium under flowing nitrogen gas. The reaction flask was placed in a pre-heated oil bath set at 90 ºC and the reaction continued for 4 hours. Upon completion the crude polymer mixture was diluted with THF and precipitated in methanol to get the solid polymer. However, a short alumina pipette column for removal of trace copper catalyst from the reaction media could be a good exercise for the students. Thus, this step was added prior to the precipitation of the crude mixture into methanol. A step-by-step instruction for short alumina pipette column is shown in **Figure S12**. The synthesized PS was characterized by GPC, shown in **Figure S13**.

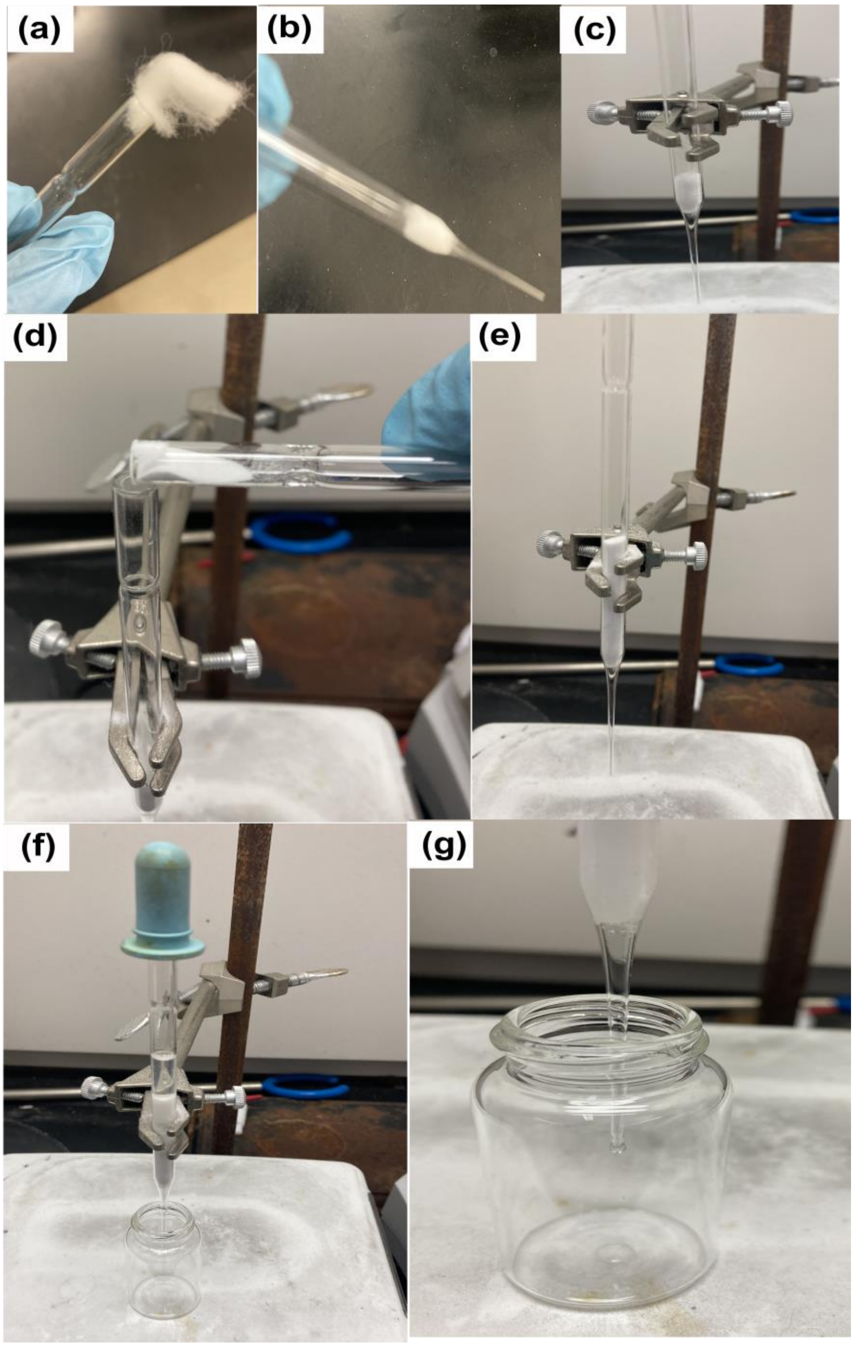

Figure S12. A piece of cotton was inserted into a glass pipette tip (a-b) and secured to a ring stand with a three-fingered clamp (c). The cotton should be placed tightly (moderate) so that liquid can pass through it, but not any solid. Alumina was scooped into the end of another pipette column (d) and poured into the clamped pipette column (e). (**Note 1**: ADJUST THE HEIGHT OF ALUMINA COLUMN DEPENDING ON THE AMOUNT OF POLYMER SOLUTION). Next, the column was moistened with THF followed by adding diluted crude polymer mixture through it (f). A dropper bulb was attached (f) to apply gentle air pressure to facilitate the pushing of the sample through the column (**Note 2**: THE POLYMER SOLUTION VISCOSITY/DILUTION SHOULD BE ADJUSTED SO THAT IT DOES NOT CLOG THE COLUMN). Collect the Cu free liquid in a separate scintillation vial/beaker (g).

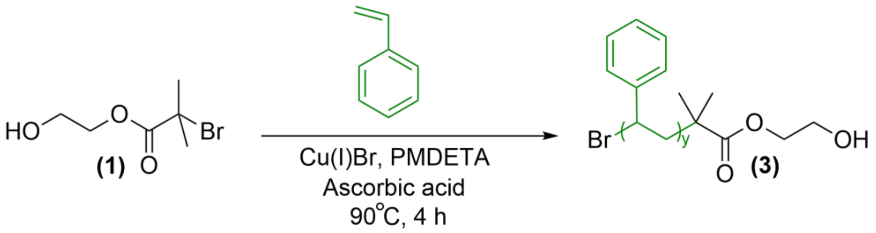

Default Individual Report

SAMPLE INFORMATION

Sample Name:	PS-P	Acquired By:	System
Sample Type:	Broad Unknown	Sample Set Name:	240206
Vial:	4	Acq. Method Set:	THF_1mLmin
Injection #:	1	Processing Method:	PS Standards
Injection Volume:	80.00 ul	Channel Name:	410
Run Time:	45.0 Minutes	Proc. Chnl. Descr.:	RI
Date Acquired:	2/6/2024 4:02:51 PM EST		
Date Processed:	2/7/2024 3:05:10 PM EST		

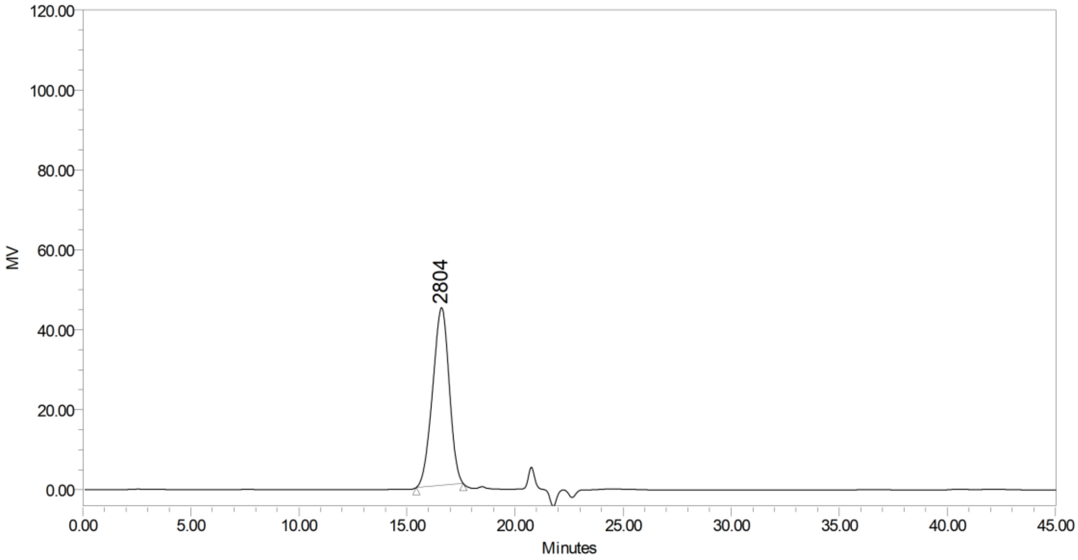

	Peak Name	RT	Area	% Area	Height
1	Broad	16.604	2366638	100.00	44445

GPC Results

	Dist Name	Mn	Mw	MP	Mz	Mz+1	Mv	Polydispersity	MW Marker 1	MW Marker 2
1		2719	3144	2804	3639	4190		1.156311		

Figure S13. GPC of the PS synthesized using PMDETA ligand and ascorbic acid reducing agent. The reaction was conducted for 4 hours.

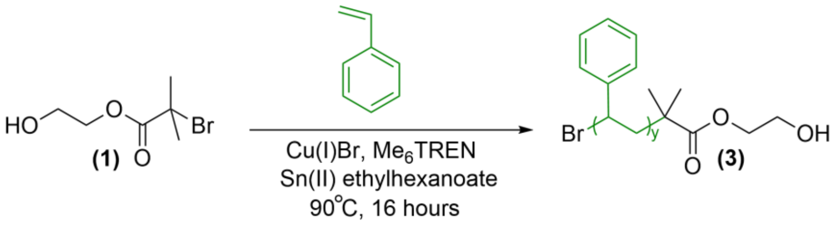

Empower™ 3 SOFTWARE

Default Individual Report

SAMPLE INFORMATION

Sample Name:	PS-M	Acquired By:	System
Sample Type:	Broad Unknown	Sample Set Name:	240206
Vial:	3	Acq. Method Set:	THF_1mLmin
Injection #:	1	Processing Method:	PS Standards
Injection Volume:	80.00 ul	Channel Name:	410
Run Time:	45.0 Minutes	Proc. Chnl. Descr.:	RI
Date Acquired:	2/6/2024 3:16:40 PM EST		
Date Processed:	2/7/2024 3:04:52 PM EST		

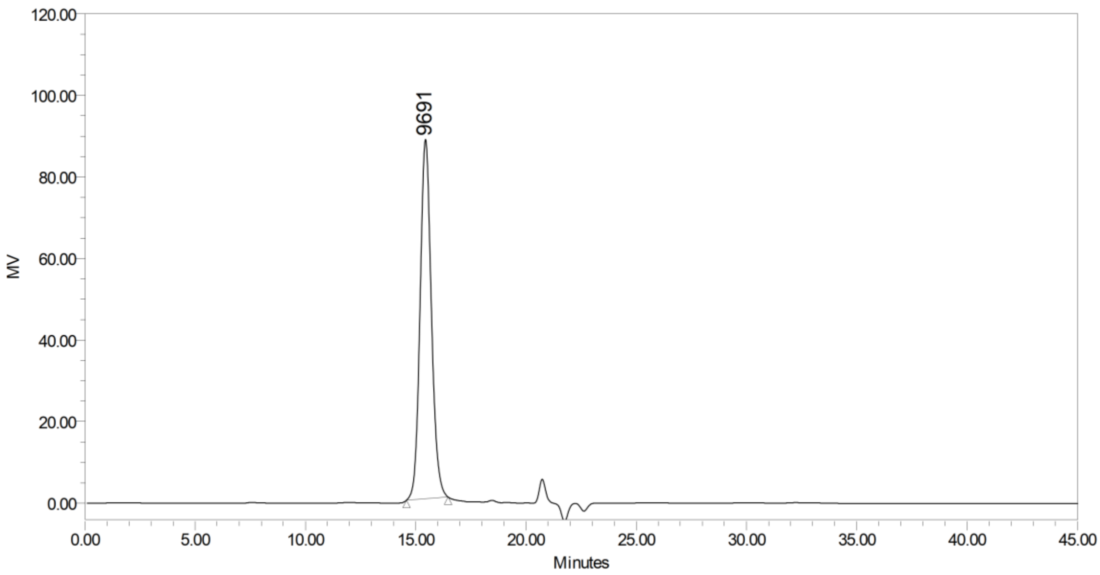

	Peak Name	RT	Area	% Area	Height
1	Broad	15.445	3118790	100.00	88139
2	Peak5	20.960			

GPC Results

	Dist Name	Mn	Mw	MP	Mz	Mz+1	Mv	Polydispersity	MW Marker 1	MW Marker 2
1		9065	9719	9691	10380	11062		1.072131		
2										

Figure S14. GPC of a PS synthesized using Me_6TREN ligand and tin(II)ethyl hexanoate reducing agent, and the polymerization was conducted for 16 hours.
